# A Trade-Off for Maintenance of Multidrug-Resistant IncHI2 Plasmids in Salmonella enterica Serovar Typhimurium through Adaptive Evolution

**DOI:** 10.1128/msystems.00248-22

**Published:** 2022-08-30

**Authors:** Jin-Fei Zhang, Liang-Xing Fang, Man-Xia Chang, Ming Cheng, Hui Zhang, Teng-Fei Long, Qian Li, Xin-lei Lian, Jian Sun, Xiao-Ping Liao, Ya-Hong Liu

**Affiliations:** a National Risk Assessment Laboratory for Antimicrobial Resistance of Animal Original Bacteria, College of Veterinary Medicine, South China Agricultural University, Guangzhou, China; b Guangdong Provincial Key Laboratory of Veterinary Pharmaceutics Development and Safety Evaluation, South China Agricultural University, Guangzhou, China; c Guangdong Laboratory for Lingnan Modern Agriculture, Guangzhou, Guangdong, China; d Jiangsu Co-Innovation Center for the Prevention and Control of Important Animal Infectious Diseases and Zoonoses, Yangzhou University, Yangzhou, China; University of California, Irvine

**Keywords:** IncHI2 plasmid, *Salmonella*, fitness cost, adaptive evolution, plasmid stability, compensatory mutation

## Abstract

Understanding the fitness costs associated with plasmid carriage is a key to better understanding the mechanisms of plasmid maintenance in bacteria. In the current work, we performed multiple serial passages (63 days, 627.8 generations) to identify the compensatory mechanisms that Salmonella enterica serovar Typhimurium ATCC 14028 utilized to maintain the multidrug-resistant (MDR) IncHI2 plasmid pJXP9 in the presence and absence of antibiotic selection. The plasmid pJXP9 was maintained for hundreds of generations even without drug exposure. Endpoint evolved (the endpoint of evolution) S. Typhimurium bearing evolved plasmids displayed decreased growth lag times and a competitive advantage over ancestral pJXP9 plasmid-carrying ATCC 14028 strains. Genomic and transcriptomic analyses revealed that the fitness costs of carrying pJXP9 were derived from both specific plasmid genes and particularly the MDR regions and conjugation transfer region I and conflicts resulting from chromosome-plasmid gene interactions. Correspondingly, plasmid deletions of these regions could compensate for the fitness cost that was due to the plasmid carriage. The deletion extent and range of large fragments on the evolved plasmids, as well as the trajectory of deletion mutation, were related to the antibiotic treatment conditions. Furthermore, it is also adaptive evolution that chromosomal gene mutations and altered mRNA expression correlated with changed physiological functions of the bacterium, such as decreased flagellar motility, increased oxidative stress, and fumaric acid synthesis but increased Cu resistance in a given niche. Our findings indicated that plasmid maintenance evolves via a plasmid-bacterium adaptative evolutionary process that is a trade-off between vertical and horizontal transmission costs along with associated alterations in host bacterial physiology.

**IMPORTANCE** The current idea that compensatory evolution processes can account for the “plasmid paradox” phenomenon associated with the maintenance of large costly plasmids in host bacteria has attracted much attention. Although many compensatory mutations have been discovered through various plasmid-host bacterial evolution experiments, the basis of the compensatory mechanisms and the nature of the bacteria themselves to address the fitness costs remain unclear. In addition, the genetic backgrounds of plasmids and strains involved in previous research were limited and clinical drug resistance such as the poorly understood compensatory evolution among clinically dominant multidrug-resistant plasmids or clones was rarely considered. The IncHI2 plasmid is widely distributed in Salmonella Typhimurium and plays an important role in the emergence and rapid spread of its multidrug resistance. In this study, the predominant multidrug-resistant IncHI2 plasmid pJXP9 and the standard Salmonella Typhimurium ATCC 14028 bacteria were used for evolution experiments under laboratory conditions. Our findings indicated that plasmid maintenance through experimental evolution of plasmid-host bacteria is a trade-off between increasing plasmid vertical transmission and impairing its horizontal transmission and bacterial physiological phenotypes, in which compensatory mutations and altered chromosomal expression profiles collectively contribute to alleviating plasmid-borne fitness cost. These results provided potential insights into understanding the relationship of coexistence between plasmids encoding antibiotic resistance and their bacterial hosts and provided a clue to the adaptive forces that shaped the evolution of these plasmids within bacteria and to predicting the evolution trajectory of antibiotic resistance.

## INTRODUCTION

Antibiotics have been a major accomplishment of modern medicine, but these compounds have suffered a loss of efficacy due to the emergence and dissemination of resistance among bacterial pathogens (1, 2). Therefore, novel strategies are needed to ensure clinical efficacy and to effectively curb the development and spread of bacterial resistance. Bacterial plasmids encode a wide range of phenotypic traits that allow bacteria to adapt to stressors such as the presence of antibiotics and have a key role in bacterial ecology and evolution (3). Interestingly, plasmids are maintained in bacterial populations over the long term, even in the absence of selection for plasmid-encoded traits. This “plasmid paradox” remains poorly understood, since plasmid carriage itself incurs physiological fitness costs to the host bacterium (4). Therefore, understanding the adaptive forces that shape the evolution of these plasmids within a bacterial host may explain the widespread distribution and stable maintenance of plasmids. This information could be used to predict the evolutionary trajectory of antibiotic resistance (5).

Salmonella is an important zoonotic pathogen, and currently, multidrug-resistant (MDR) strains that are resistant to fluoroquinolones, third-generation cephalosporins, and even colistin, such as Salmonella enterica serovar Typhimurium and its variants, have emerged (6, 7). Incompatibility HI2 (IncHI2) plasmids are often large (>200 kb) with similar backbone structures and possess two sets of conjugation systems along with numerous antibiotic resistance genes (ARGs) (8, 9). These IncHI2 plasmids are widespread among MDR *Enterobacteriaceae* and especially S. Typhimurium, even though they incur a high fitness cost for their maintenance (8–10).

In this study, we performed 63 serial passages to explore the compensatory mechanisms of adaptive evolution of the MDR IncHI2 plasmid pJXP9 within S. Typhimurium ATCC 14028 in the presence and absence of antibiotics. Our findings suggest that plasmid maintenance through plasmid-host bacterial coadaptation and coevolution is a trade-off but that coevolution of this large IncHI2 plasmid promoted host cell growth and competitiveness. This coevolution thereby increased plasmid vertical transmission by increasing host competitiveness and decreased growth lag times but impaired horizontal transmission of the plasmid and altered host bacterial physiology in a given niche. These results provide insights into understanding the mechanisms that bacteria use to offset the costs of stable plasmid maintenance.

## RESULTS

### Reducing lag time and improving competition advantage in evolved bacteria through plasmid-bacterium coadaptation.

**FIG 1:**
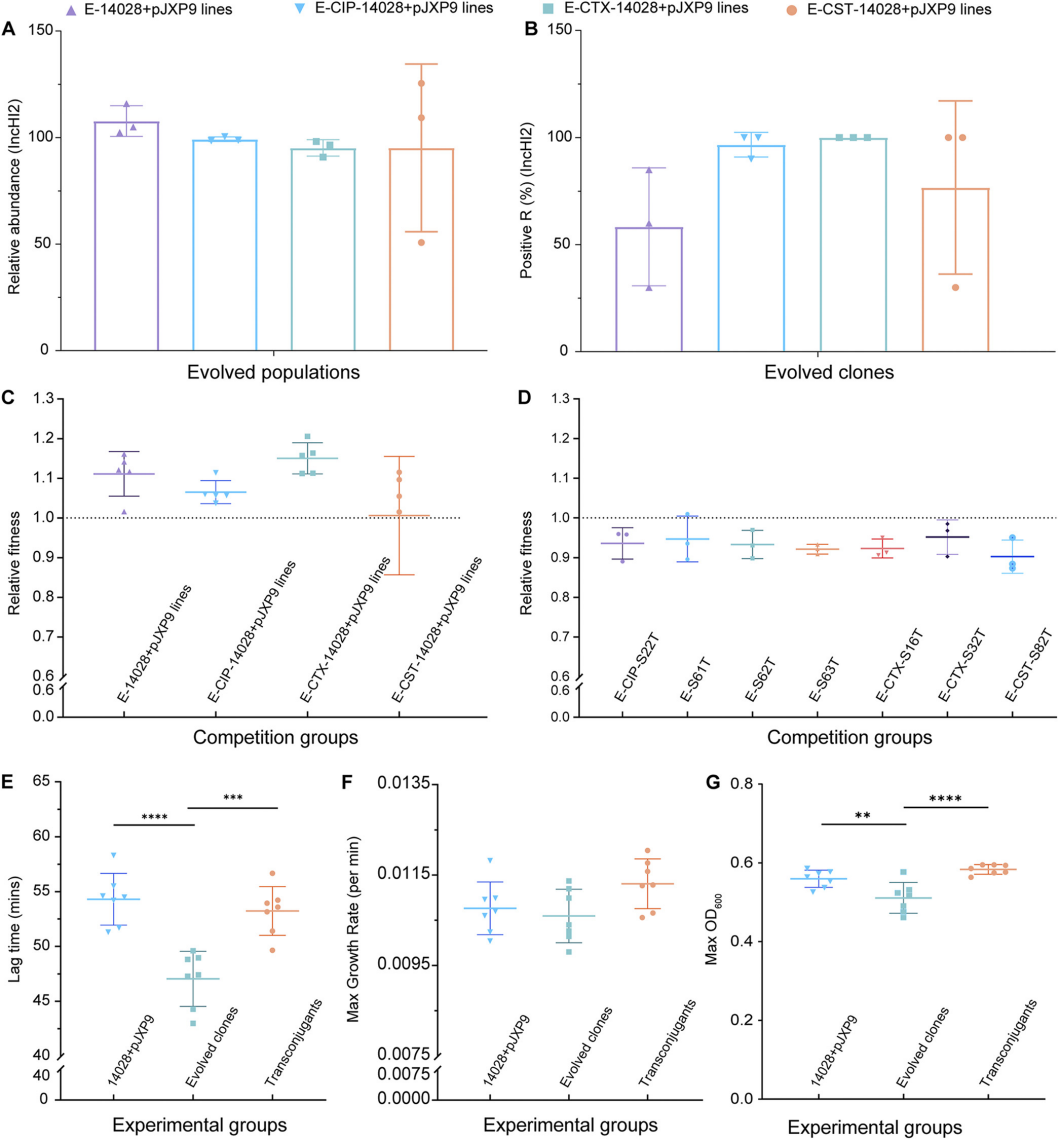
Percentage abundances of *repHI2* gene and fitness costs of plasmid carriage in evolved populations and clones. (A) Relative abundance of *repHI2* gene in evolved populations. (B) Detection of *repHI2* gene in evolved clones. (C) Relative fitness of 20 evolved ATCC 14028 clones bearing evolved pJXP9 versus ATCC 14028::*lux*+pJXP9 by competition assays. (D) Relative fitness of 7 ancestral ATCC 14028 clones bearing evolved pJXP9 (transconjugants) versus ATCC 14028::*lux*+pJXP9 by competition assays. (E to G) Lag time (E), growth rates (F), and culture density (max OD_600_ values) (G) were calculated for 7 evolved clones bearing evolved pJXP9, corresponding transconjugants, and ATCC 14028::*lux*+pJXP9. All data from at least three biological replicates are presented as the mean ± SD, and the significance was determined by nonparametric one-way ANOVA (*, *P < *0.05; **, *P < *0.01; ***, *P < *0.001; ****, *P < *0.0001).

The experimental system we used for this study was a comparison of the cost of maintaining a large IncHI2 plasmid (pJXP9) in S. Typhimurium strain ATCC 14028 with that of its plasmid-free counterpart. Preliminary experiments indicated that carriage of pJXP9 impaired competition and generated a slight growth disadvantage for strain ATCC 14028 (see Fig. S1 in the supplemental material). We extended these experiments and performed serial dilutions of cultures over 63 days in the presence and absence of antibiotics to investigate the stability of the IncHI2 plasmid in endpoint evolved clones/populations (clones/populations from the endpoint of evolution). Using PCR for 240 endpoint evolved clones and quantitative real-time PCR (qPCR) for 12 endpoint evolved populations, we found that IncHI2 plasmid pJXP9 was retained at day 63 with almost no loss when cultured in the presence of ciprofloxacin (CIP) and cefotaxime (CTX) but that plasmid levels were slightly reduced in the presence of colistin (CST) (24% loss using PCR and 7.7% loss using qPCR screening). Interestingly, culture in the absence of antibiotics led to significant plasmid loss (41.7% loss using PCR and 52.3% loss using qPCR screening) (Fig. 1A and B; Fig. S2). These results indicate that carriage of pJXP9 imposed a fitness cost, but the plasmid was maintained for at least hundreds of generations even in the absence of positive selection. To determine the fitness response to plasmid-bacterial host coevolution with or without antibiotic treatment, we directly competed 5 randomly selected endpoint evolved clones from each treatment (total, 20) against strain ATCC 14028::*lux*+pJXP9, which possessed a luciferase fluorescent marker integrated into the host chromosome. We found an increase in the competitive fitness in all 20 endpoint evolved clones (relative fitness [RF] range, 1.015 to 1.206) except one (RF = 0.7482) (Fig. 1C). These results revealed that adaptive evolution had occurred between the IncHI2 plasmid pJXP9 and the host, ATCC 14028.

10.1128/msystems.00248-22.1 FIG S1 Estimating the fitness effects of pJXP9 plasmid carriage. (A) Growth of ancestral strain ATCC 14028 with the pJXP9 plasmid. (B) Relative fitness of pJXP9 plasmid-carrying versus plasmid-free ancestral ATCC 14028 using competition assays. (C and D) Growth of ancestral ATCC 14028 versus ATCC 14028::*lux* for plasmid-free (C) and pJXP9-carrying (D) strains. Download FIG S1, TIF file, 0.7 MB. Copyright © 2022 Zhang et al. 2022 Zhang et al. https://creativecommons.org/licenses/by/4.0/ This content is distributed under the terms of the Creative Commons Attribution 4.0 International license.

10.1128/msystems.00248-22.2 FIG S2 Gene abundance analysis by qPCR of clinically important ARGs among endpoint evolved populations. Download FIG S2, TIF file, 1.2 MB. Copyright © 2022 Zhang et al. 2022 Zhang et al. https://creativecommons.org/licenses/by/4.0/ This content is distributed under the terms of the Creative Commons Attribution 4.0 International license.

To establish whether the fitness alterations were due to evolution of the plasmid, the bacterial host, or both, the evolved pJXP9 plasmids from evolved hosts were transferred to host ATCC 14028::*lux*, and 7 transconjugants carrying evolved plasmids were successfully obtained. The competitive fitness measurements for all these transconjugants remained less than those of ATCC 14028+pJXP9 (RF range, 0.88 to 1.01) (Fig. 1D). We further quantified the fitness levels for the 7 evolved clones and corresponding transconjugants using growth curves. Compared to the 7 transconjugants, the corresponding evolved clones displayed significant reductions in growth lag times (47.05 versus 54.30 min) (*P < *0.05) (Fig. 1E) but also a decreased growth rate (0.01059 versus 0.01076 per min) (*P > *0.05) (Fig. 1F) and a decreased maximum optical density at 600 nm (max OD_600_) (0.5109 versus 0.5594) (*P < *0.05) (Fig. 1G). In contrast, compared to the ancestral host ATCC 14028+pJXP9, the corresponding transconjugants of these evolved plasmids possessed higher growth rates (0.01131 versus 0.01076 per min) (*P > *0.05) (Fig. 1F) and max OD_600_ values (0.5833 versus 0.5594) (*P > *0.05) (Fig. 1G) and decreased lag times (53.23 versus 54.30 min) (*P > *0.05) (Fig. 1E). Taken together, these findings suggest that evolution of both the plasmid and the host contribute to fitness effect changes in endpoint evolved clones. The competitive advantage and reduced lag times were primarily due to the evolved host, while carriage of the evolved pJXP9 plasmid slightly improved the growth rate and maximum culture density but impaired the competitive fitness.

### Diversity in large fragment deletions among plasmid MDR and conjugative transfer regions on evolved isolates.

**FIG 2:**
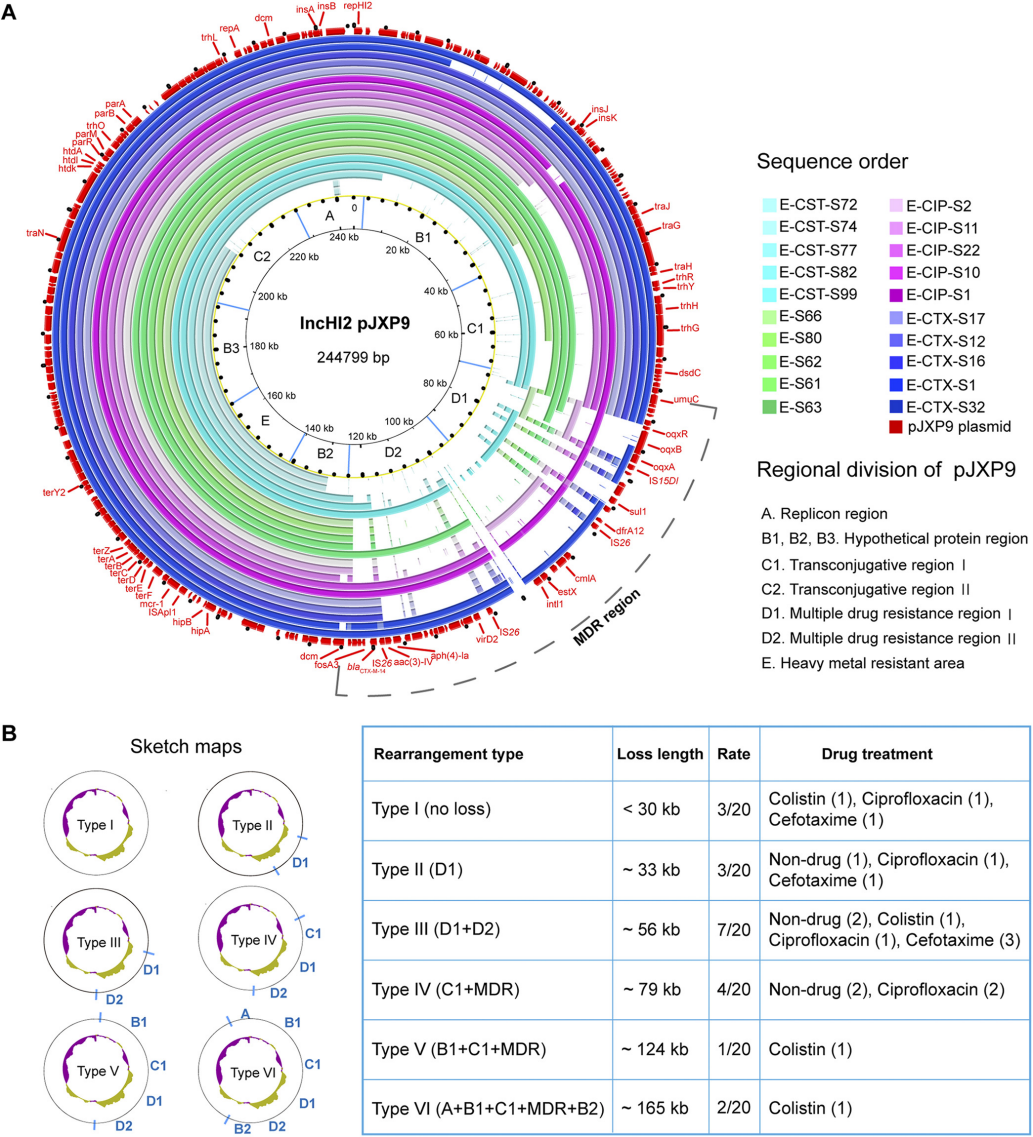
Sequence analysis of endpoint evolved pJXP9 plasmids. (A) Circular sequence alignment of 20 evolved pJXP9 plasmids from endpoint evolved clones with ancestral pJXP9 plasmid from this study created using the BLAST Ring Image Generator (BRIG). Colored circles from outside to inside correspond to the list (sequence order) in the legend. Regional division of pJXP9 is according to the function of genes in the ancestral pJXP9 plasmid. (B) Summary information of deletion profiles for endpoint evolved plasmid pJXP9 from 20 selected evolved clones.

To determine the contribution of pJXP9 plasmid evolution to cost alleviation, whole-genome sequencing (WGS) data of the pJXP9 plasmids from 20 endpoint evolved clones using 4 different antibiotic conditions were utilized to identify any gene deletions or additions that may have occurred during endpoint evolution experiments (Fig. 2A; Fig. S3). In general, the derived plasmids ranged in size from ~80 to ~244 kb, and the GC content tended to decline (see figure at https://doi.org/10.6084/m9.figshare.20416359). We identified six types of deletions and rearrangements. Type I included 3 clones with deletions of <30-kb fragments. The other five deletion types included the following plasmid regions: MDR region I (MDR I) (*n* = 3, type II), MDR region (*n* = 7, type III), MDR I and MDR region II (MDR II) and conjugative transfer region I (*n* = 4, type IV), MDR I and II and conjugative transfer region I (*n* = 1, type V), and an ~165-kb region including *repHI2* (*n* = 2, type VI), which was found in two evolved clones, E-CST-S72 and E-CST-S74. Interestingly, deletion sizes were related to antibiotic selection as follows: CST, range of ~20 to ~165 kb; CTX, ~20 to ~50 kb; CIP, ~30 to ~90 kb; and no antibiotic, ~30 to ~79 kb (Fig. 2B). These results indicated a diversity of large fragment deletions for the plasmid MDR and conjugative transfer regions.

10.1128/msystems.00248-22.3 FIG S3 Sequence comparison of evolved plasmid pJXP9 from 10 evolved clones sequenced by Illumina and Nanopore. Sequence comparison of evolved pJXP9 with ancestral pJXP9 as the reference was conducted using the BLAST Ring Image Generator (BRIG) v0.95. The same color series indicates that the plasmids are derived from clones under the same exposure condition. Download FIG S3, TIF file, 2.9 MB. Copyright © 2022 Zhang et al. 2022 Zhang et al. https://creativecommons.org/licenses/by/4.0/ This content is distributed under the terms of the Creative Commons Attribution 4.0 International license.

### Gene relative abundance is reduced in plasmid MDR and conjugative transfer regions in evolved populations.

**FIG 3:**
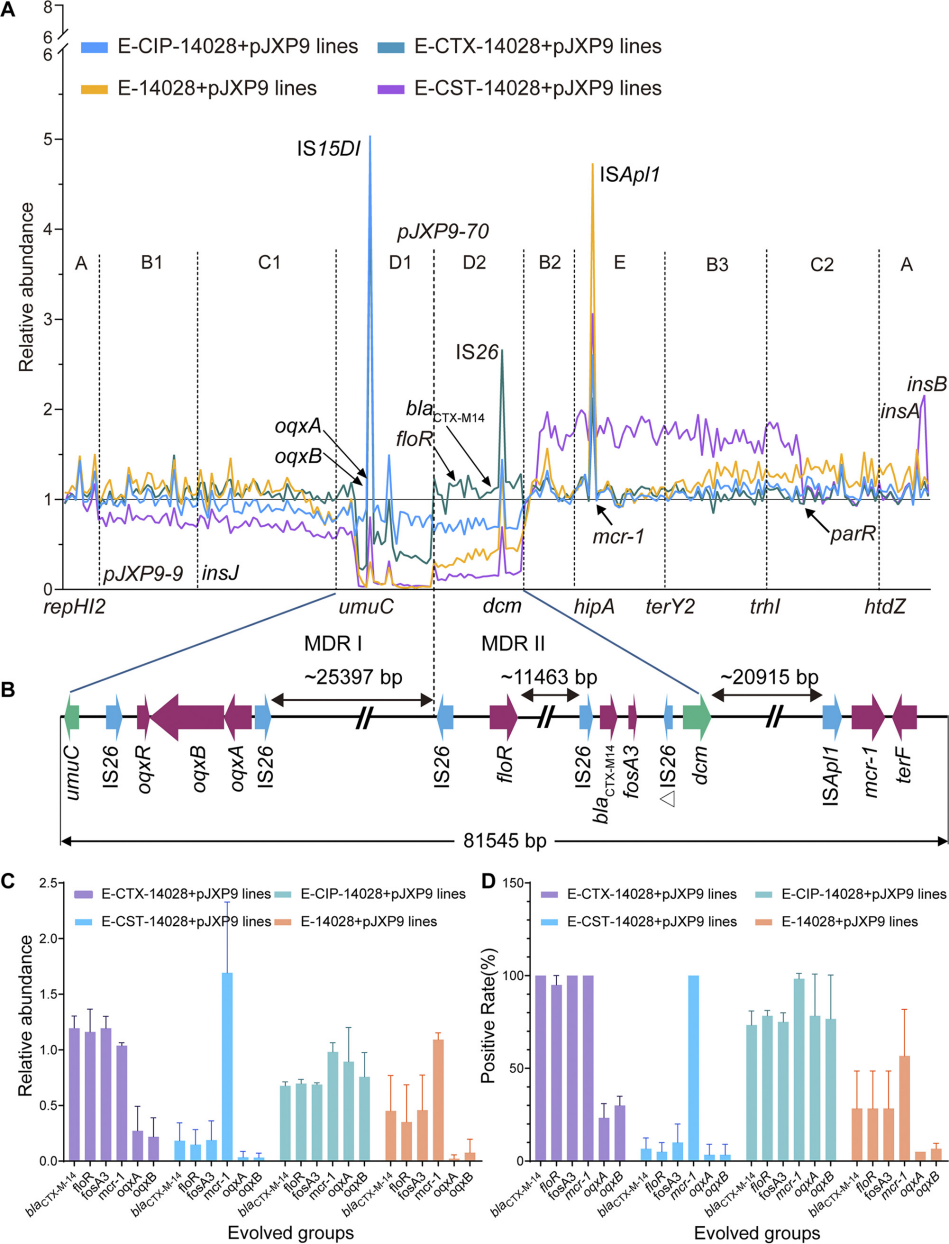
Relative abundance of genes in plasmid pJXP9 from evolved populations under 4 experimental culture conditions. (A) Distribution of gene abundance in plasmid pJXP9 from endpoint evolved populations. (B) Characteristics of the MDR regions in ancestral pJXP9. (C) Relative abundance for clinically important ARGs in endpoint evolved populations using population sequencing. (D) Detection rates of clinically important ARGs among endpoint evolved clones from evolved populations under 4 exposure conditions. All data were derived from at least 3 biological replicates and are presented as the mean ± SD, and levels of significance were determined by nonparametric one-way ANOVA (*, *P < *0.05; **, *P < *0.01; ***, *P < *0.001; ****, *P < *0.0001).

We further determined the relative abundance in evolved IncHI2 plasmid pJXP9 by using the WGS data for 12 endpoint evolved populations under different treatments compared with ancestral plasmid pJXP9 populations. We found that alterations had occurred primarily in the MDR regions between *umuC* and *dcm* (~56 kb), including MDR I containing *oqxAB* between *umuC* and *pJXP9-70* (~33 kb) (D1) and MDR II containing *floR*, *bla*_CTX-M-14_, and *fosA3* between *pJXP9-70* and *dcm* (~23 kb) (D2) (Fig. 3A and B). Notably, the relative abundance of MDR I and MDR II depended on the treatments: MDR I was retained at >60% with CIP and >25% with CTX, but gene loss was ~100% with CST and no antibiotic. In contrast, MDR II was retained at 100% with CTX, 65% with CIP, and 25% with no antibiotic and at 15% with CST. Regions (~85 kb, B2 + E + B3) containing *mcr-1*, *ter*-like genes, and hypothetical protein genes between *dcm* and *parR* largely increased in relative abundance to ~150% but declined to ~70% in region I containing the conjugative transfer region I (C1, ~38 kb) between *insJ* and *umuC* and a large hypothetical protein region (B1, ~56 kb) between *pJXP9-9* and *umuC* under CST treatment. Furthermore, relative abundances were slightly decreased to ~89% in the conjugative transfer region I (C1) under CIP treatment. However, the relative abundance was slightly increased to ~120% in the regions (~76 kb, between *terY2* and *insA/insB*) containing a hypothetical protein region (B3, ~23 kb) between *terY2* and *trhI* and the conjugative transfer region II (C2, ~36 kb) between *trhI* and *htdZ* in the absence of antibiotics (Fig. S4).

10.1128/msystems.00248-22.4 FIG S4 Relative abundances of genes in plasmid pJXP9 from evolved populations during 63 serial passages under ciprofloxacin and nondrug exposure. (A) Relative abundance of genes in plasmid pJXP9 under ciprofloxacin exposure. E-CIP-1, -14, -28, -42, or -63 denotes populations selected at day 1, 14, 28, 42, or 63 under ciprofloxacin exposure, respectively. (B) Relative abundance of genes in plasmid pJXP9 under nondrug exposure. E-nondrug-1, -14, -28, -42, or -63 denotes populations selected at day 1, 14, 28, 42, or 63 under nondrug exposure, respectively. Download FIG S4, TIF file, 2.6 MB. Copyright © 2022 Zhang et al. 2022 Zhang et al. https://creativecommons.org/licenses/by/4.0/ This content is distributed under the terms of the Creative Commons Attribution 4.0 International license.

Additionally, gene relative abundances were largely increased for several insertion sequences, including IS*15DI* (containing 3 mutated codons compared with IS*26*) adjacent to *oqxA* and IS*Apl1* adjacent to *mcr-1* and IS*26* adjacent to *bla*_CTX-M14_. Intriguingly, the increased extent of gene relative abundance for these insertion sequences largely depended on treatment conditions. For example, the gene relative abundance of IS*15DI* was increased under CIP and CTX treatments, whereas IS*Apl1* relative abundance was largely increased in the absence of antibiotics, followed by that under CST, CIP, and CTX treatments. The gene relative abundances for IS*26* increased under CTX, followed by that under CIP and no treatment. The gene relative abundance remained almost stable in the other regions or genes on evolved plasmids under different treatments compared with ancestral plasmid pJXP9-carrying populations. Taken together, these results were consistent with the sequence analysis of pJXP9 from endpoint evolved clones in which the MDR and conjugative transfer regions were frequently deleted in evolved plasmids and these deletions depended on antibiotic treatment conditions.

### The stability of clinically relevant ARGs carried by evolved plasmids was antibiotic treatment dependent.

The stability of clinically relevant ARGs in endpoint evolved clones and populations was further investigated using PCR (240 clones), qPCR (12 populations), and WGS (12 populations) analyses (Fig. 3C and D; Fig. S2). We found that the stability of *mcr-1*, *bla*_CTX-M-14_, *fosA3*, *oqxAB*, and *floR* was highly variable and differed between antibiotic treatments. Under CIP treatment, all five tested ARGs remained at high relative abundance, with detection rates of >70% and *mcr-1* abundance at ~100%. CTX treatment resulted in high abundance in all tested ARGs but *oqxAB*, which was retained at ~25%. Unexpectedly, under CST treatment, *mcr-1* was completely retained but all of the other four ARGs were almost completely lost at a rate of ~90% and *oqxAB* was lost at ~100%. Additionally, serial transfer in the absence of antibiotic selection resulted in retention of *mcr-1* at ~80%, followed by that of *bla*_CTX-M-14_, *fosA3*, and *floR* at ~45%. In contrast, *oqxAB* was almost completely lost (loss rate, ~95%). Antimicrobial susceptibility testing also indicated that resistance phenotypes of the 240 evolved clones were consistent with the presence of *oqxAB*, *mcr-1*, *bla*_CTX-M-14_, *fosA3*, and *floR* (Table S3). Taken together, these results indicate that similar to the MDR regions deletions, the stability of clinically relevant ARGs carried by evolved plasmids were also antibiotic treatment dependent.

10.1128/msystems.00248-22.9 TABLE S3 Summary of MIC and PCR data for all 240 evolved clones in evolution experiments. Download Table S3, DOCX file, 0.02 MB. Copyright © 2022 Zhang et al. 2022 Zhang et al. https://creativecommons.org/licenses/by/4.0/ This content is distributed under the terms of the Creative Commons Attribution 4.0 International license.

### Different antibiotic treatments altered the trajectory of deletion mutations in evolved plasmids.

To determine the order of the large fragment deletion mutations in evolved plasmids, we investigated the temporal dynamics of pJXP9 and ATCC 14028 coadaptation evolution under ciprofloxacin and nondrug treatments by WGS using time series-evolved populations (days 1, 14, 28, 42, and 63) (Fig. S4). Under CIP exposure, genes in MDR I (excluding insertion sequences) were almost completely maintained and were stable across 63 serial passages, while those in MDR II (excluding insertion sequences) and C1 decreased to ~70% at day 63. Unexpectedly, the region containing *oqxR*-*oqxA*-*oqxB* and the class I integron harboring *aad1* (aminoglycoside resistance)-*cmlA* (chloramphenicol resistance)-*estX* (streptothricin acetyltransferase) were completely lost by day 42 and returned to the normal levels found in the ancestral population at day 63. In contrast, passages in the absence of antibiotics generated a gradual decline of MDR II that was slight at days 14 and 28 and more pronounced at days 42 and 63. MDR I also showed a slight decrease at days 14 and 28 and then decreased sharply at day 42 and was completely lost by day 63. Furthermore, the partial conjugative transfer region I (between *traJ* and *umuC*) showed slight decreases at days 42 and 63 (Fig. S4). Taken together, for pJXP9 populations evolved under CIP, the class I integration region and *oqxR*-*oqxA*-*oqxB* began to be deleted at least at day 42 and were then followed by deletions of MDR II and C1 by day 63. In contrast, for pJXP9 populations evolved under the absence of drug selection, large fragment deletions first occurred in MDR I and were then followed by deletions in MDR II and the partial conjugative transfer region I. Furthermore, the abundance of IS*15DI* (adjacent to *oqxA*), IS*26* (adjacent to *bla*_CTX-M14_), and IS*Apl1* (adjacent to *mcr-1*) tended toward increasing, and the temporal dynamics was also observed under CIP and nondrug treatments.

### Mutations of chromosomal genes involved in the stress response contributed to the competition advantage of evolved hosts.

**FIG 4:**
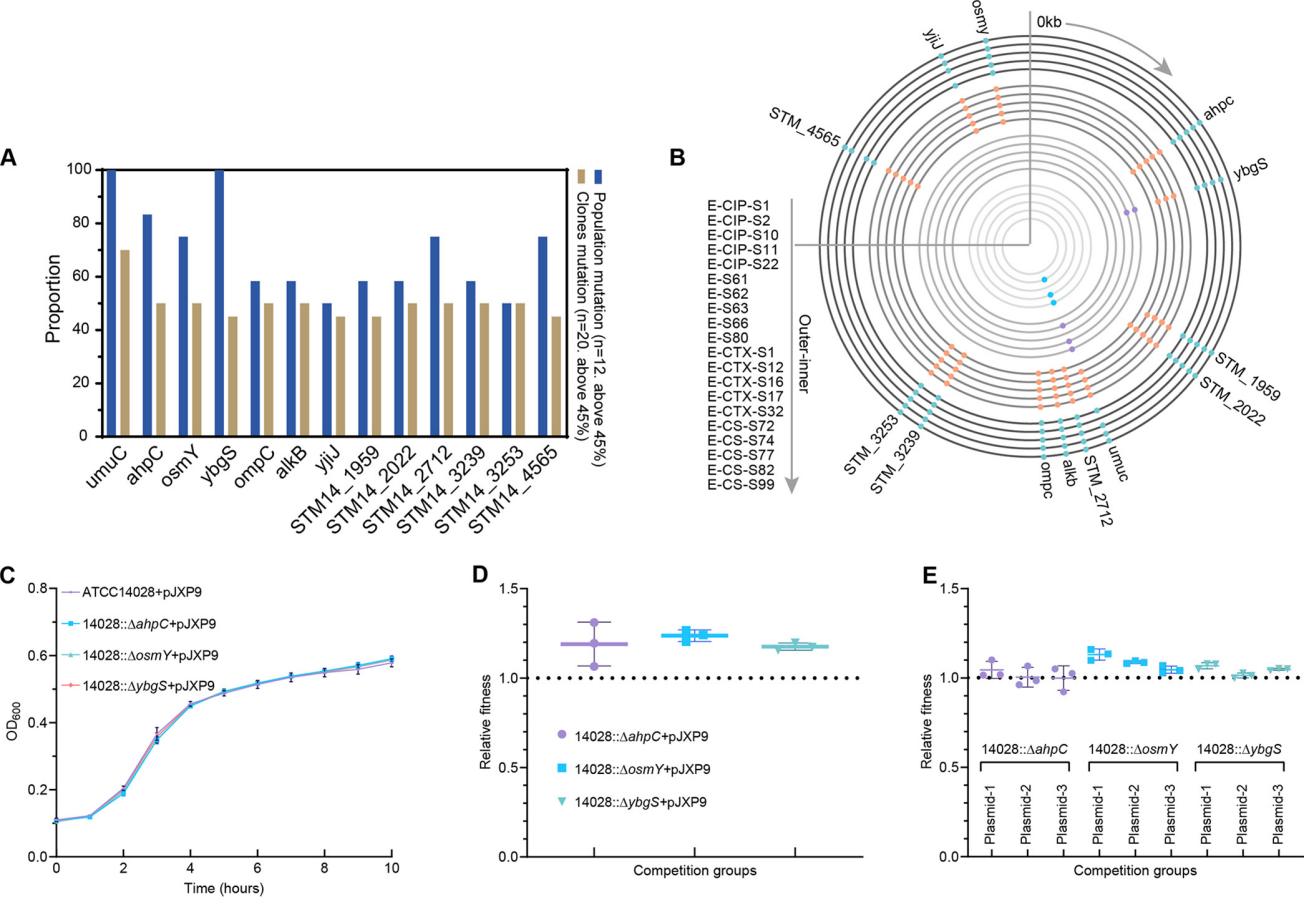
Chromosomal compensatory mutation analysis and function verification. (A) Frequency analysis of compensatory mutations in chromosomal genes in populations and clones. Mutant frequencies were defined by numbers of occurrences in 12 evolved populations or 20 representative clones. The mutated genes also included mutations at different sites. Cutoffs were set at >0.45, because mutated genes were among the top 25% simultaneously in populations and clones. *n*, number of populations or individual clones used to detect mutations. (B) Mutants present in 20 evolved clones. Different colors represent different exposure conditions. (C) Growth curves for mutated ATCC 14028+pJXP9 and ancestral ATCC 14028+pJXP9 strains. (D) Relative fitness of mutated ATCC 14028+pJXP9 versus ATCC 14028::*lux*+pJXP9 by competition assays. (E) Fitness estimation of mutated ATCC 14028 carrying evolved plasmid pJXP9 versus ATCC 14028::*lux*+pJXP9 strains. Plasmids 1 (from E-S62), 2 (from E-S63), and 3 (from E-CTX-S32) represent three types of evolved pJXP9 plasmids, respectively. All data were derived from at least 3 biological replicates and are presented as the mean ± SD, and levels of significance were determined by nonparametric one-way ANOVA (*, *P < *0.05; **, *P < *0.01; ***, *P < *0.001; ****, *P < *0.0001).

To analyze whether putative chromosomal modifications mitigate the fitness costs of pJXP9 plasmid carriage, we determined the complete genome sequences of 20 endpoint evolved clones, 12 endpoint evolved populations, and 2 ancestral clones as described above, as well as 8 controls (14,028 evolved clones/population). A total of 81 shared mutated chromosomal genes were identified after excluding mutations also found in the ancestral clones and controls. The mutations identified were common in 13 genes, with frequencies of ≥45% among both multiple evolved clones and populations. These genes were primarily associated with oxidative stress (*ahpC*, *ybgS*, *STM14_1959*, and *STM14_2022*), DNA repair (*umuC* and *alkB*), outer membrane permeability (*ompC*), osmotic stress (*osmY*), and sugar transporter (*yjiJ*), as well as hypothetical proteins (*STM14_2712*, *STM14_3239*, *STM14_3253*, and *STM14_4565*) (Fig. 4A and B). The gene *ahpC* encodes an alkyl hydroperoxide reductase subunit belonging to a two-cysteine peroxiredoxin family and is responsible for protecting cells from low concentrations of H_2_O_2_ (11). The hypothetical protein YbgS containing two cysteine residues was probably associated with redox reactions (12). *STM14_1959* and *STM14_2022* encode a putative oxidoreductase and oxidase, respectively. These two genes act together with *ahpC* and *ybgS* as antioxidant-related genes, and inactivation of these genes most likely alters the ability of the oxidative stress response. AlkB is an alkylated DNA repair protein (13). DNA polymerase V subunit gene *umuC* is directly involved in the induction of mutagenesis and associated with the SOS response (14, 15). Mutations in these two DNA repair genes could enhance mismatch repair. The outer membrane porin C protein OmpC allows for ions and hydrophilic solutes to cross the outer membrane, and its mutation could lead to impairment of the structural integrity and change in the permeability of the outer membrane (13). Molecular chaperone OsmY is associated with osmotic shock and the growth state of bacteria (16–19), and mutation inactivation of this gene could impair osmotic stress and result in delayed growth compared with that of wild-type S. Typhi in SPI-2-inducing conditions (17). YjiJ is a putative sugar transporter, and its mutation might be associated with glucose metabolic disorders.

To directly test whether loss of gene function plays a role in ameliorating the cost of plasmid carriage, *ahpC*, *osmY*, and *ybgS* were selected, and knockout mutants of these genes were constructed in the ancestral ATCC 14028 background carrying ancestral and evolved plasmid pJXP9. We measured the fitness relative to the wild type with plasmid pJXP9. We found that with Δ*ahpC*, Δ*osmY*, or Δ*ybgS*, ATCC 14028 mutants carrying ancestral plasmid pJXP9 had higher competitive advantages and slightly lower growth rates than the ancestral strain ATCC 14028+pJXP9 (Fig. 4C and D). Furthermore, Δ*ahpC*, Δ*osmY*, or Δ*ybgS* mutants carrying evolved pJXP9 also exhibited slightly higher competitive advantage than ancestral ATCC 14028+pJXP9 (Fig. 4E). This indicated that these gene deletions could generally alleviate the cost produced by possession of pJXP9 to some extent. Furthermore, multiple gene mutations were identified in endpoint evolved populations or clones that were under CIP or nondrug exposure (Fig. 4B). Taken together, adaptive coevolution of plasmid pJXP9 within S. Typhimurium ATCC 14028, particularly in the presence of CIP or with no drug, promoted the competitiveness of the evolved bacterial host through compensatory mutations in primarily stress response genes.

### Alterations in convergent gene mRNA abundance were related to impaired physiological functions in evolved bacterial hosts with rearranged pJXP9 plasmids.

**FIG 5:**
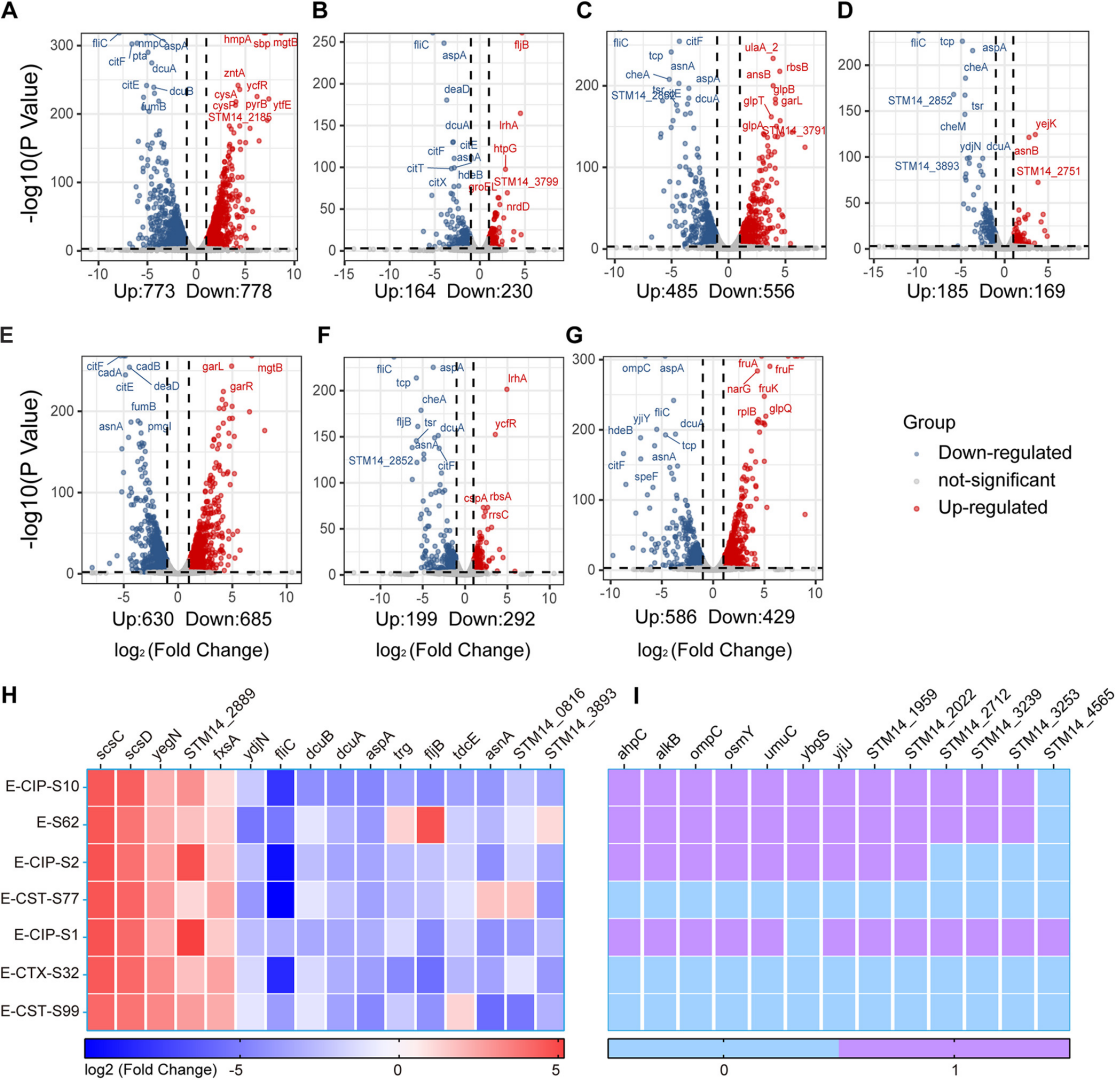
Transcriptome profiles for 7 representative endpoint evolved clones relative to that of the ancestral ATCC 14028+pJXP9 without antibiotic pressure. (A) E-CIP-S10 (type II, D1 deletion); (B) E-S62 (type III, MDR region deletion); (C) E-CIP-S2 (type IV, MDR region + C1 deletion); (D) E-CST-S77 (type V, MDR region + B1 + C1 deletion); (E) E-CIP-S1 (type I, no loss); (F) E-CTX-S32 (type I, no loss); (G) E-CST-S99 (type I, no loss). The 7 endpoint evolved clones were selected based on the deleted regions of evolved plasmid pJXP9 in Fig. 2. The colored points define significant (***, *P < *0.001) differential expression (>2-fold change). Points represent upregulated genes (light red), downregulated genes (light blue), and not-significant genes (gray), respectively. (H) Shared up- or downregulated genes in seven evolved isolates. (I) Shared mutated genes in seven evolved isolates.

Phenotype and genome analyses of evolved populations and clones indicated that the carriage costs of pJXP9 plasmid in Salmonella could be ameliorated through mutations in chromosomal genes associated with the stress response and large fragment deletions in the conjugative transfer regions and multidrug resistance regions in the plasmid. This suggested that there were convergent physiological responses to the carriage of and compensation with this plasmid. To understand these responses and their resolution, we performed RNA sequencing (RNA-seq) on endpoint evolved isolates carrying nearly complete plasmid pJXP9 (3 isolates) and incomplete ones with different deleted regions, from ~33 kb to ~165 kb (4 isolates) (Fig. 5A to G), with or without chromosomal amelioration mutations (Fig. 5I). The pJXP9 plasmid-carrying ancestral clone was chosen as a control. In these 7 evolved isolates, chromosomal gene expression was altered, and the numbers of significantly upregulated and downregulated genes ranged from 185 to 773 and from 169 to 778, respectively (Fig. 5A to G). Furthermore, the number of significantly downregulated genes was slightly higher than that of upregulated genes in 5/7 evolved isolates. The opposite situation was observed in the remaining 2 isolates (Fig. 5D and G). Notably, a set of 16 shared chromosomal differentially expressed genes (DEGs) were identified in all of these 7 evolved clones. These included 5 upregulated genes (*scsC*, *scsD*, *yegN*, *STM14_2889*, and *fxsA*) and 5 downregulated genes (*ydjN*, *fliC*, *dcuB/dcuA*, and *aspA*), as well as 6 genes that were up- or downregulated in different evolved isolates (Fig. 5H). AspA is linked to the tricarboxylic acid (TCA) cycle and aspartate metabolic pathways and catalyzes the conversion of fumarate to l-aspartate (20). *dcuA*/*dcuB* encodes common C4-dicarboxylate/aspartate transporters that are active under anaerobiosis (20, 21). The downregulation of these 3 genes (AspA, dcuA and dcuB) indicated that aspartate metabolic pathways might be inhibited. Interestingly, the fimbrial gene *fliC*, which encodes a structural flagellar protein (22), was downregulated, and this would reduce flagellar motility. YdjN functions as a transporter of S-sulfocysteine, a sulfur-containing intermediate in assimilatory cysteine biosynthesis (23), and its downregulation might impair cystine metabolism and oxidative stress responses (24). *scsABCD* encode 4 proteins in Salmonella that resemble the disulfide folding machinery of other bacteria, and upregulation of *scsBC* is linked to adaptation to Cu and H_2_O_2_ stress (25). *yegN* is part of a tripartite efflux system, and its deletion results in decreased growth (26). FxsA overproduction in Escherichia coli inhibits the F plasmid-mediated exclusion of bacteriophage T7 and interacts with the F plasmid-encoded PifA protein to minimize membrane damage (27).

Our transcriptome data from evolved plasmid pJXP9, which remained intact from evolved isolates E-CIP-S1, E-CTX-S32, and E-CST-S99, revealed that plasmid-borne genes located on the conjugative transfer regions (C1 and C2) were almost all downregulated except for *parAB* and *parMR*. Almost all of the MDR genes were upregulated, with a few exceptions (Fig. S5). Of note, the corresponding resistance phenotypes in these three clones were almost not changed (Table S3). Taken together, the gene expression alterations in sets of shared chromosomal and plasmid-borne genes in the endpoint evolved clones would most likely result in impaired fumaric acid synthesis, flagellar motility, and antioxidant activity, as well as decreased conjugation and bacteriophage-mediated horizontal gene transfer but enhanced resistance against copper.

10.1128/msystems.00248-22.5 FIG S5 Alterations in mRNA abundance in MDR region (A) and transferable regions (B and C) of pJXP9 from evolved clones bearing complete evolved plasmid pJXP9 against ancestral ATCC 14028 carrying pJXP9. Download FIG S5, TIF file, 0.9 MB. Copyright © 2022 Zhang et al. 2022 Zhang et al. https://creativecommons.org/licenses/by/4.0/ This content is distributed under the terms of the Creative Commons Attribution 4.0 International license.

## DISCUSSION

**FIG 6:**
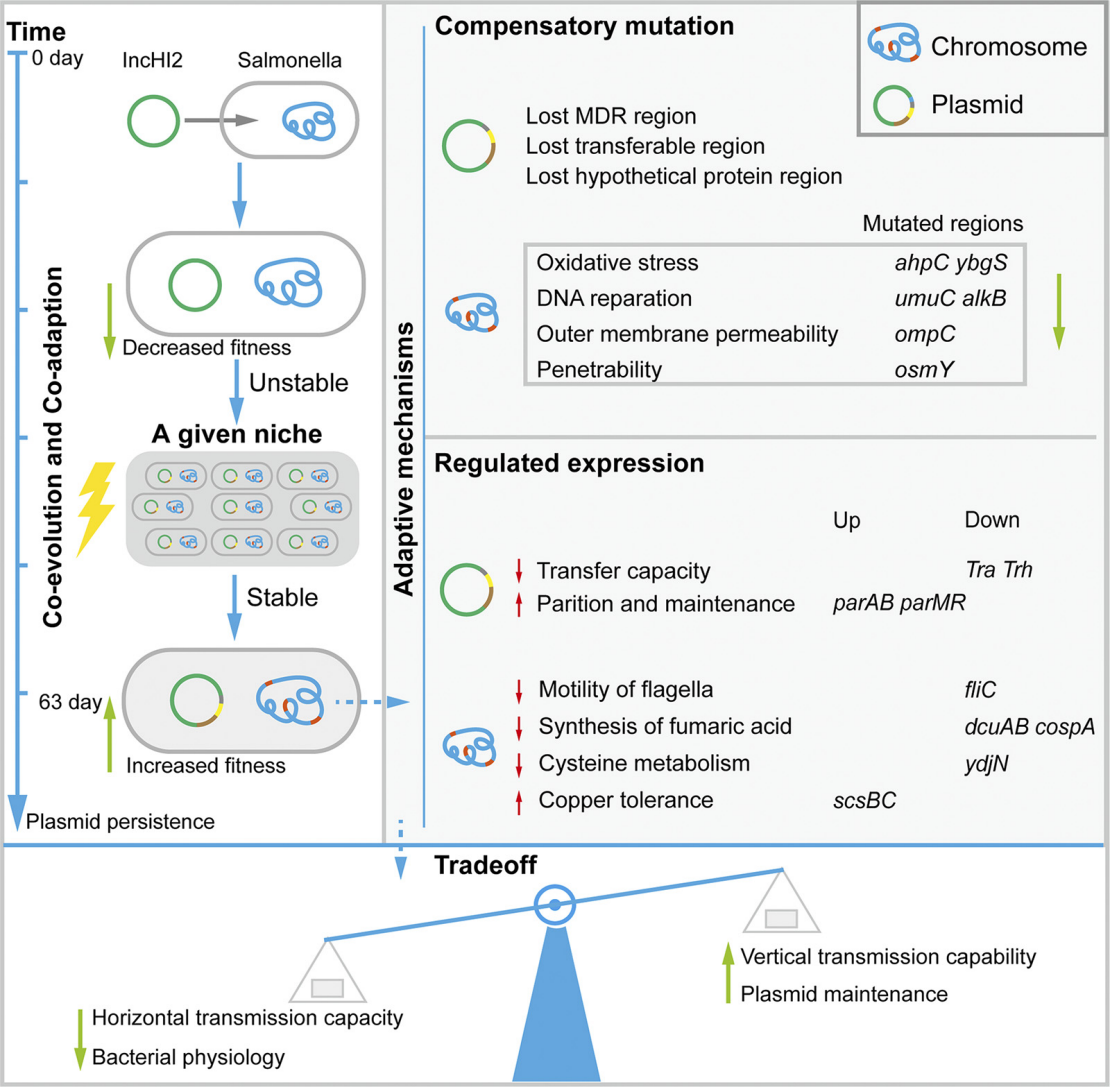
Possible mechanisms underlying adaptive evolution of the IncHI2 plasmid pJXP9 and bacterial host Salmonella Typhimurium ATCC 14028.

IncHI2 plasmids are relatively large (>200 kb), and how they are successfully maintained or persist in the bacterial host is unknown, in particular in S. Typhimurium. We found that the fitness cost imposed on S. Typhimurium ATCC 14028 by IncHI2 plasmid pJXP9 carriage was partially alleviated through coculture of IncHI2 plasmids and S. Typhimurium ATCC 14028. Specifically, experimental evolution was performed for hundreds of generations to achieve potential mutation or fitness-improved information (28). These compensatory mutations included deletions in the plasmid MDR and conjugative transfer regions and specific hypothetical protein regions in evolved plasmids (Fig. 6). Interestingly, these changes were dependent of the presence and type of antibiotic, and similar scenarios of large plasmid MDR region deletions have been reported for evolved plasmid pKP33 in Klebsiella pneumoniae and evolved plasmid pUR2940 in Staphylococcus aureus lineages (5, 29). Indeed, these deletions that we found in the MDR region enhanced the growth rate of transconjugants compared with that of ancestral pJXP9-carrying clones. Furthermore, the evolved isolates E-CIP-S1, E-CTX-S32, and E-CST-S99 carried evolved plasmid pJXP9 without any large fragment deletion, while almost all gene expression from transconjugative regions I and II were downregulated except for that of *parAB* and *parMR* (see Fig. S5 in the supplemental material). Conjugation is energetically expensive for host cells, and impairing conjugative transfer probably benefits plasmid maintenance in the bacterial host (30). *parAB* and *parMR* are inserted into the transfer gene clusters and capable of supporting partitioning in IncHI2 plasmids (31), and since these genes were not downregulated, their expression most likely facilitated plasmid partitioning and maintenance in the bacterial host. However, unexpectedly, increased expression of MDR genes was observed on evolved plasmid pJXP9 from evolved isolates E-CIP-S1, E-CTX-S32, and E-CST-S99, although MDR region deletions were also common in other evolved clones or evolved populations. This process was most likely linked to an increase in survival ability under antibiotic exposure. Taken together, the conjugation transfer and MDR regions seemed to be one of the primary factors that exerted fitness costs on carriage of IncHI2 plasmids while adaptive plasmid evolution includes deletions that promoted host bacterial growth while retaining the plasmid.

We also identified numerous parallel compensatory mutations that appeared in evolved plasmids that included many types of fragment deletions, and interestingly, the extent and range of these deletions on the plasmid in endpoint evolved populations could be linked to culture conditions. Similarly, the differentiated trajectory of deletion mutations in evolved plasmids was also found to be related to selection pressure during the serial evolutionary process under ciprofloxacin and nondrug exposure. But the reasons why gene abundance and deletion mutations occurred between drug exposure groups at specific times remain unclear. One possible explanation is that insertion sequences (ISs) most likely were the arbiters of fragment deletion and recombination (29, 32), and this was reflected in the high gene relative abundance of IS elements in evolved populations. This was especially true for *oqxAB*, *bla*_CTX-M14_, and *mcr-1* under corresponding antibiotic treatment conditions. Indeed, ISs contribute to ARG transmission in response to antibiotic exposure and are involved in plasmid-bacterium coadaptation (33–35). Taken together, these results illustrated the dynamics and complexity of plasmid-bacterium coadaptation and coevolution under different antibiotic treatments and time points. The ISs played a significant role in IncHI2 plasmid evolution. Specific parallel compensatory mutations or mutation trajectories were probably due to the directional selection of specific treatments with time, and it made plasmid carriage advantageous.

In addition to compensatory deletion mutations in evolved plasmids, bacterial hosts have evolved diverse chromosomal compensatory mutations, such as the global regulatory system genes *gacA*/*gacS*, the DNA helicase genes *uvrD* and *xpd/rad3*, the nonselective outer membrane porin gene *ompF*, the efflux pump genes *acrAB*/*acrR*, and PFLU4242 of unknown function (36–39). We found a series of chromosomal mutations in stress response genes in evolved clones and, in particular, under CIP exposure or the lack of a drug. We identified deletions in oxidative stress-associated genes, such as the alkyl hydroperoxide reductase gene *ahpC* and other related but uncharacterized genes (*ybgS*, *STM14_1959*, and *STM14_2022*) and genes for DNA repair (*umuC* and *alkB*), outer membrane permeability (*ompC*), and osmotic stress (*osmY*). The deletions of *ahpC*, *osmY*, and *ybgS* in the ancestral ATCC 14028 background carrying plasmid pJXP9 improved the competitive capacity of the host. Furthermore, other downregulated genes were independent of antibiotic exposure and were related to bacterial physiological functions, including impaired flagellar motility (*fliC*), blocked fumaric acid synthesis (*dcuB/A* and *aspA*), decreased resistance to oxidative stress (*ydjN*), inhibited bacteriophage-mediated horizontal gene transfer (*fxsA*), and enhanced Cu resistance (*scsCD*). Plasmid acquisition is often associated with reduced bacterial motility and is sometimes due to downregulation of flagellar genes (40). In this study, the swimming zones were significantly inhibited in endpoint evolved strains compared to those of ancestral strains that carried pJXP9 (*P < *0.01), whereas the swimming zones in transconjugants carrying evolved plasmids were restored and comparable to those of ancestral strains with ancestral pJXP9 (Fig. S6; see also Text S1 in the supplemental material). These data indicate that impaired flagellar motility was most likely the result of the evolved chromosome. This also implied that potential conflicts between plasmid-bearing and chromosomal genes were also a significant source of fitness cost of carrying pJXP9. As a result, both chromosomal compensatory mutations and altered chromosomal expression profiles influenced bacterial physiology phenotypes to reduce plasmid-carrying costs through coevolution. These indicated that plasmid fitness costs caused by specific genetic conflicts are unlikely to act as a long-term barrier for the persistence of plasmids (36). Furthermore, plasmids can also manipulate chromosomal gene expression (40). Therefore, further studies are required to determine how adaptive coevolution links these bacterial phenotypes to plasmid fitness.

10.1128/msystems.00248-22.6 FIG S6 Comparison of swimming motilities among ancestral and evolved clones and transconjugants by swimming motility experiments. (A) Comparison of swimming motilities between seven picked endpoint evolved clones carrying evolved plasmid pJXP9 and ancestral ATCC 14028 bearing pJXP9. (B) Comparison of swimming motilities between seven picked endpoint evolved clones carrying evolved plasmid pJXP9 and its corresponding transconjugants (ancestral ATCC 14028 carrying evolved plasmid pJXP9). Download FIG S6, TIF file, 0.8 MB. Copyright © 2022 Zhang et al. 2022 Zhang et al. https://creativecommons.org/licenses/by/4.0/ This content is distributed under the terms of the Creative Commons Attribution 4.0 International license.

10.1128/msystems.00248-22.10 TEXT S1 Supplemental methods. Download Text S1, DOCX file, 0.03 MB. Copyright © 2022 Zhang et al. 2022 Zhang et al. https://creativecommons.org/licenses/by/4.0/ This content is distributed under the terms of the Creative Commons Attribution 4.0 International license.

There are other ecological and evolutionary solutions to the plasmid paradox besides compensatory evolution (41). For example, chromosomal adaptive mutations controlling the global regulatory systems for carbon catabolite repression (CCR) and anaerobic metabolism (ArcAB) reduced the fitness cost of MDR plasmid carriage in specific bacterial niche adaptations (42). Furthermore, bacterial communities that contain multiple species can act as surrogate hosts to maintain a plasmid in the population (42). Moreover, an additional explanation for the maintenance of conjugative plasmids has been proposed, i.e., that plasmid donor cells can also be effective competitors with plasmid-free cells particularly in structured environments (28). Further study is required to determine whether other ecological and evolutionary mechanisms of plasmid stability, such as piggybacking on niche adaptation, can alter the maintenance of IncHI2 plasmids in S. Typhimurium.

This study proposes that the source of fitness costs of transferable MDR IncHI2 plasmids depends on plasmid gene expression as well as conflicts between these regions and the chromosome. As a result, compensatory plasmid-borne and chromosomal mutations and altered chromosomal expression profiles collectively contributed to alleviating the fitness costs during adaptative coevolution of the plasmid and the host bacterium. Of note, this adaptive coevolution is also a trade-off between improved growth and competitiveness, with impairments in bacterial physiological processes such as flagellar motility, horizontal gene transfer, cell membrane permeability, fumaric acid synthesis, and resistance to oxidative and osmotic stress. Considering that the specifics of evolutionary dynamics likely vary with the environment and organism, further studies are still required to reveal the nature of adaptive evolution of plasmids and bacteria.

## MATERIALS AND METHODS

### Strains and experimental evolution.

**FIG 7:**
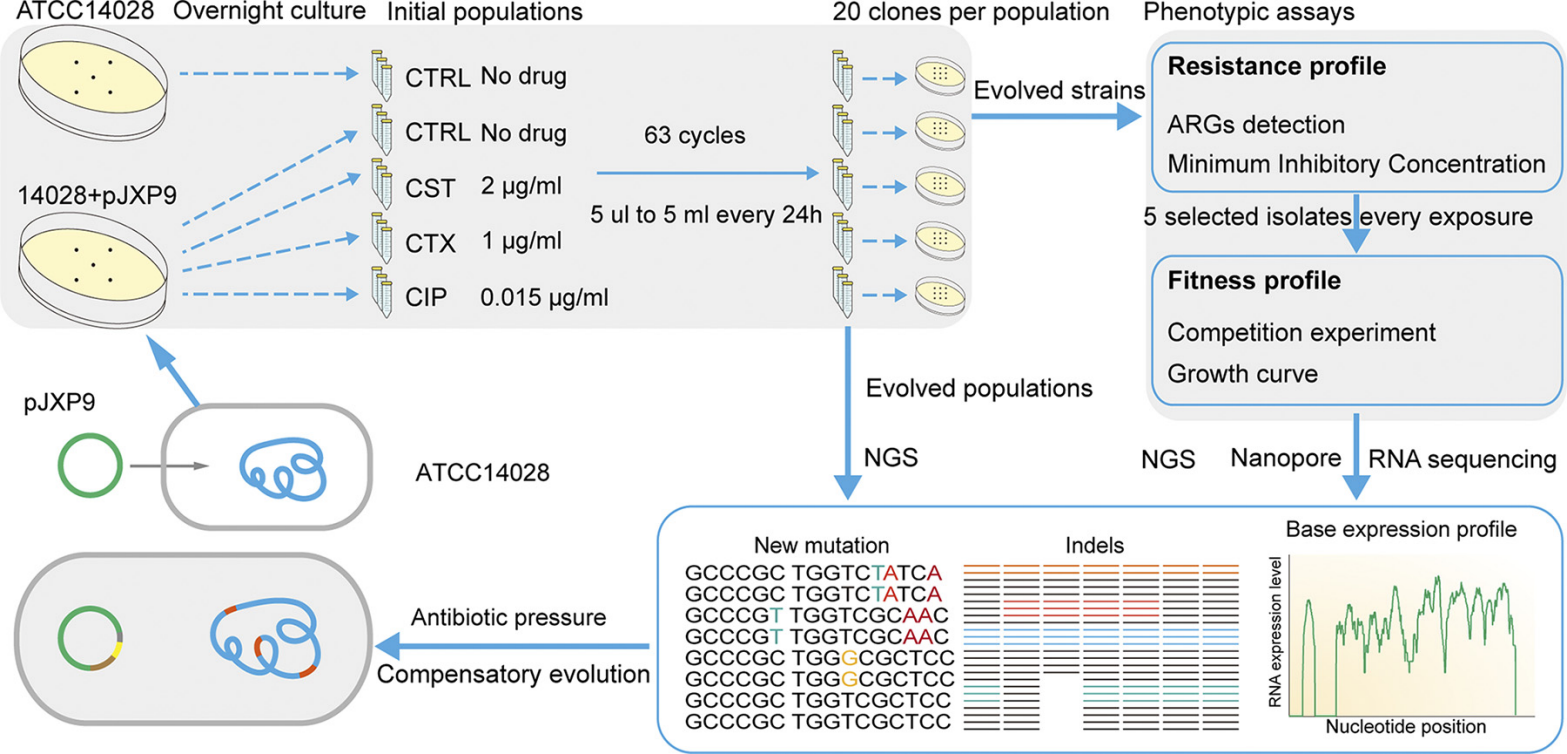
Schematic overview for adaptive evolution of plasmid-bacterial host under subinhibitory or nondrug exposure. Colonies taken from LB agar plates of ancestral clones ATCC 14028+pJXP9 and ATCC 14028 were picked and inoculated into fresh LB broth as initial cultures and serially passaged in triplicate for 63 days. Twenty clones from each evolved population were selected for MICs and ARG detection, and representative clones (*n* = 5) were selected for estimating plasmid fitness effects by competition assays and growth curves. Evolved populations and evolved clones were further submitted to WGS by Illumina. Nanopore sequencing was also performed for representative clones harboring evolved IncHI2 plasmids of different sizes. Furthermore, evolved clones carrying plasmids of different sizes were subjected to whole-genome RNA sequencing. Parallel mutations for evolved populations and clones and mRNA abundance alterations were analyzed.

The plasmid pJXP9 (~244 kb) was derived from an E. coli J53 transconjugant of S. Typhimurium JXP9 recovered from a pig in Jiangxi, China, in 2017, possessed a typical IncHI2 plasmid backbone, and carried at least 15 ARGs, including *mcr-1*, *bla*_CTX-M-14_, *fosA3*, *oqxAB*, and *floR* (9). The plasmid pJXP9 was introduced into S. Typhimurium ATCC 14028 by conjugation, and transconjugants were defined as the initial ancestral strain (denoted ATCC 14028+pJXP9) and used for experimental evolution (for details, see Text S1 in the supplemental material). Briefly, cultures were grown in 5 mL Luria Bertani (LB) broth in 50-mL tubes at 37°C and shaken at 180 rpm. Independent selection lines were founded using 12 independent single colonies of ATCC 14028+pJXP9 taken from plate cultures, grown overnight in LB in the absence of antibiotics (nonselective conditions), and split into four exposure groups with biological triplicates: (i) no antibiotic selection, (ii) 2 μg/mL colistin (CST), (iii) 1 μg/mL cefotaxime (CTX), and (iv) 0.015 μg/mL ciprofloxacin (CIP). In parallel, three independent ATCC 14028 colonies were picked up for control treatments and grown in the absence of selection. Cultures were serially diluted 1/1,000 every consecutive day (24 h) for 63 days (63 × log_2_ 1,000) to achieve ~627.8 generations calculated as previously described (28, 43). The final tally for these experiments was 15 endpoint evolved populations (5 exposure scenarios and biological triplicates for each exposure) and a total of 300 endpoint clones (20 clones were selected randomly from each evolved population), which were stored in 30% glycerol at −80°C and used later for phenotyping, whole-genome sequencing (WGS), and RNA sequencing (Fig. 7). Additionally, evolved populations derived from days 1, 14, 28, and 42 that included 3 populations had been passaged in the absence of antibiotics, and 3 populations in the presence of CIP were also stored at −80°C and further deep sequenced (see below).

### Plasmid stability, ARGs, and MIC detection.

The stability of pJXP9 and its ARG content were determined at the end of the selection experiment using PCR screening of 20 randomly picked colonies from each tested endpoint population (240 total) using primer sets specific for the plasmid genes *repHI2*, *mcr-1*, *bla*_CTX-M-14_, *oqxAB*, *fosA3*, and *floR* (Table S1). Plasmid drug resistance was assessed by determining the MICs for 6 antibiotics, including colistin (CST), florfenicol (FFC), cefotaxime (CTX), fosfomycin (FOS), ciprofloxacin (CIP), and nalidixic acid (NAL), in the selected colonies (total, 300 endpoint clones), and the results were interpreted according to the CLSI and veterinary CLSI guidelines (44, 45). MICs for FOS were determined using Mueller-Hinton agar supplemented with 25 μg/mL glucose-6-phosphate. Escherichia coli ATCC 25922 served as the quality control strain.

10.1128/msystems.00248-22.7 TABLE S1 PCR primers used in this study. Download Table S1, DOCX file, 0.03 MB. Copyright © 2022 Zhang et al. 2022 Zhang et al. https://creativecommons.org/licenses/by/4.0/ This content is distributed under the terms of the Creative Commons Attribution 4.0 International license.

The relative abundances of the 6 plasmid target genes in endpoint populations were determined using quantitative real-time PCR (qPCR) as previously described (2). Briefly, total DNA was extracted from evolved and ancestral pJXP9-bearing populations that were grown in LB broth at 37°C for 10 h. qPCR assays were performed in triplicate using the primers listed in Table S1. The 16S rRNA gene was used as an internal control for DNA quantification. Relative quantification was calculated using the 2^−ΔΔ^*^CT^* method (46) [ΔΔ*C_T_* = (*C_T_*, _target_ − *C_T_*, _control_)_evolved populations_ − (*C_T_*, _target_ − *C_T_*, _control_)_ancestral populations_].

### Growth curves and competitive fitness assays.

Growth curves and competition assays are frequently used methods for estimating plasmid fitness effects (47). Growth curves were measured using triplicate overnight cultures that were diluted to an OD_600_ of 0.1 in LB broth and distributed in 96-well plates at 200 μL per well. The assay plates were placed into an EnSight multimode plate reader (PerkinElmer, USA) and incubated at 37°C with continuous shaking at 180 rpm, and growth was recorded by measuring the optical density (λ = 600 nm) for a minimum of 10 h. These data were used to extract parameters that served as proxies for bacterial fitness: (i) maximum growth rate (estimating the intrinsic population growth rate), (ii) maximum optical density (max OD_600_, carrying capacity), and (iii) lag phase duration (lag time, defined as the integrated time lost during adaptation to new conditions compared with an immediate response) (48, 49). Data were processed using the Growthcurver package in R (50). Growth curves were also constructed for ancestral strain ATCC 14028 and ATCC 14028+pJXP9 as well as for endpoint clones bearing evolved plasmid (for details, see Text S1).

The relative fitness (RF) of plasmid-carrying versus plasmid-free clones or ancestral plasmid-carrying versus evolved plasmid-carrying clones was estimated using direct *in vitro* competition assays in triplicate as previously described with slight modifications (51). In brief, growth competition was initiated using a strain cultured for 24 h in LB medium at 37°C and then diluted to an OD_600_ of 0.1, mixed in a 1:1 ratio, incubated at 37°C for 24 h (day 1), and then diluted to obtain separate colonies when plated on LB agar containing 2 μg/mL CTX or no antibiotic to obtain CFU. Competition assays using ancestral ATCC 14028 versus the ancestral pJXP9-bearing ATCC 14028 strain were performed for 7 consecutive days following the described methods. Competition assays of evolved ATCC 14028 strains bearing the evolved pJXP9 versus ATCC 14028::*lux*+pJXP9 and of ancestral ATCC 14028 bearing the evolved plasmid versus ATCC 14028::*lux*+pJXP9 were performed in 1 day. Colonies were screened for epifluorescence using a Leitz Aristoplan microscope and compared to the total plate CFU. The relative fitness was calculated using the equation RF = (log_10_ *S1*_dt_ − log_10_ *S1*_d0_)/(log_10_ *S2*_dt_ − log_10_ *S2*_d0_), where *S1* and *S2* represent CFU densities of two tested clones (*t* = time in days), d0 and dt represent the time when CFU densities of clone *S1* or clone *S2* were determined respectively (51). An RF of >1 indicated a selective advantage over the control strain, whereas an RF of <1 represented a fitness cost.

### Genome sequencing and plasmid analysis.

Representative evolved clones were sequenced based on MIC and PCR results. Genomic DNA was extracted using a Gentra Puregene bacterial DNA purification kit (Qiagen, Hilden, Germany) according to the manufacturer’s instructions. The DNA was sequenced using an Illumina HiSeq platform (Novogene, China), and sufficient sequencing depth (>100) was obtained for further analysis (1 Gb per clone and 6 Gb per population). The raw data of genomes were filtered by Trimmomatic with -phred 33 (v 0.32), and the clean data were assembled to calculate the estimated genome change using SPAdes (v3.6.2) (-t 30 -k 21,33,55,77,99,127 –careful –phred-offset 33) (52) (Table S2).

10.1128/msystems.00248-22.8 TABLE S2 Detailed information for clean data and assembled contigs of 20 evolved clones. Download Table S2, DOCX file, 0.02 MB. Copyright © 2022 Zhang et al. 2022 Zhang et al. https://creativecommons.org/licenses/by/4.0/ This content is distributed under the terms of the Creative Commons Attribution 4.0 International license.

High-quality reads were mapped to the plasmid pJXP9 sequence using SOAP aligner/SOAP2 with a maximum alignment error of 5 (53). The relative gene abundances of evolved plasmids (all genes in pJXP9) from evolved populations for transfers at days 1, 14, 28, 42, and 63 were normalized using RPKM (reads per kilobase per million reads) with ancestral pJXP9 as the reference (54). Alignments between evolved plasmids and ancestral plasmids and generation of circular maps were performed using BRIG (v 0.95) (55). Nanopore sequencing was further conducted to obtain the complete nucleotide sequence of plasmid pJXP9 in endpoint evolved clones. For clones sequenced by Nanopore and Illumina, sequence assembly was performed using Unicycler using default parameters for hybrid assembly (v 0.4.8) (56). Plasmid size and GC content alterations for evolved chromosome and plasmid were compared with those of ancestral references using Snapgene (v5.0). The sizes of IncHI2 plasmids were measured from 2 ancestral and 20 evolved strains after linearization of genomic DNA using S1 nuclease (TaKaRa, Dalian, China) by pulsed-field gel electrophoresis (PFGE), followed by Southern blotting hybridization using a digoxigenin-labeled probe specific for *repHI2* (Table S1) as previously described (57) (for details, see Text S1).

### Mutation analysis and function verification.

Sequencing reads of endpoint ATCC 14028+pJXP9 evolved populations and clones were mapped to the ATCC 14028 reference genome (accession no. CP001363 downloaded from https://www.ncbi.nlm.nih.gov/nuccore/CP001363/), and basic variants were called using the CLC Genomics Workbench 10.0 (Qiagen, Hilden, Germany) with default parameters (basic variant detection, similarity fraction of 0.9, minimum coverage of 100, minimum count of 10, and minimum frequency of 10.0%). All chromosomal mutations occurring in >10% of the reads and in at least 10 unique reads were included in the analysis, while those occurring in noncoding regions were excluded (58, 59). Then, the mutations that were present in corresponding endpoint ATCC 14028 evolved populations and clones were filtered out from the above-described mutation results. To better reflect the origin of mutation among populations and clones, we clustered different mutations into the corresponding genes using the following rules: (i) mutations that occurred in >10% in either evolved populations or clones were defined as valid and (ii) cutoffs for gene mutation frequencies among evolved populations and clones were set to acquire the top 25% of the mutated genes.

To confirm the impact of chromosomal gene mutation on fitness cost of pJXP9 plasmid carriage, the selected target genes were deleted in ancestral strain ATCC 14028 bearing plasmid pJXP9/evolved plasmid pJXP9 by use of the two-plasmid system pCaspa-pSGKp (based on CRISPR/Cas9-mediated genome editing) CRISPR-Cas9 methods as described in Text S1. The designed 20-nt base-pairing region (N20) of sgRNA for deleting targeted genes and primers for detecting fragment deletion are listed in Table S1. Growth curves and competitive fitness assays were performed among the mutation strains as described above.

### Whole-genome RNA sequence analysis and bioinformatics.

In order to analyze the gene expression levels of endpoint evolved clones with evolved plasmid, RNA was extracted from the selected clones cultured in LB broth for 4 h without antibiotics as previously described (60, 61). Bacterial cells were collected by centrifugation at 8,000 × *g* for 10 min. The library was constructed using an Illumina TruSeq RNA sample prep kit v2 as previously described (62) and sequenced using the Illumina HiSeq 2000 platform. High-quality reads that passed the Illumina quality filter with Trimmomatic -phred 33 were mapped to the S. Typhimurium strain ATCC 14028 genome using the FANSe 2 algorithm with parameters -L55 -E2 -U1 -S10. Genes with ≥10 mapped reads were considered confidently detected genes. Gene expression levels were estimated using RPKM (reads per kilobase per million reads) (54). Gene differential expression analysis was performed as previously described (63). The genes with a <0.001 false discovery rate (FDR) and a change of >2-fold or <0.5-fold were detected as differentially expressed genes (DEGs).

### Statistical analyses.

Statistical analyses were performed using Prism 8.0 (GraphPad, San Diego, CA, USA). All data were obtained from at least three biological replicates and are presented as the mean ± standard deviation (SD). Unpaired Student's *t* test (nonparametric) between two groups and one-way analysis of variance (ANOVA) (nonparametric) among multiple groups with a *post hoc* test were used to calculate *P* values. Significance levels are indicated as follows: *, *P < *0.05; **, *P < *0.01; ***, *P < *0.001; and ****, *P < *0.0001.

### Additional material.

Supplementary figures that support the findings in this research were submitted to the figshare database (https://doi.org/10.6084/m9.figshare.20416359).

### Data availability.

The population sequencing data and RNA sequencing data reported in this paper have been deposited in the NCBI SRA and GEO databases (BioProject accession no. PRJNA810373 and PRJNA810452 and GEO accession no. GSE197475), respectively. Complete sequences of the evolved strains sequenced by Nanopore and Illumina have been deposited in the GenBank database under accession numbers SAMN26490219 to SAMN26490228. All other data related to this study are available upon request.
