# Supplementary figures and images for: A Trade-Off for Maintenance of Multidrug-Resistant IncHI2 Plasmids in Salmonella enterica Serovar Typhimurium through Adaptive Evolution
Source: mSystems. 2022 Aug 30;7(5):e00248-22. doi: 10.1128/msystems.00248-22 (PMC9599605; doi:10.1128/msystems.00248-22)

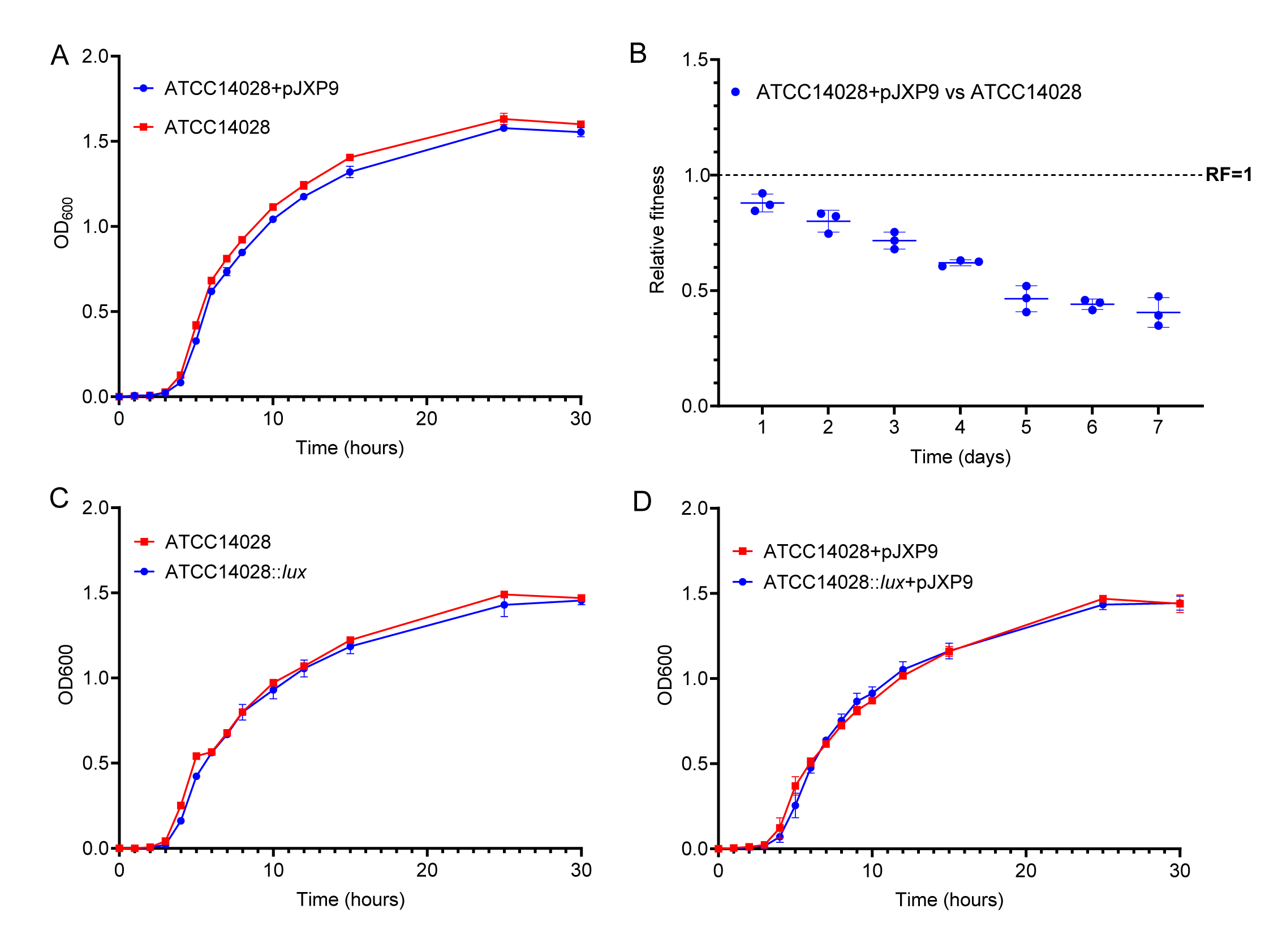

Supplement: FIG S1 [file msystems.00248-22-s0006.tif]

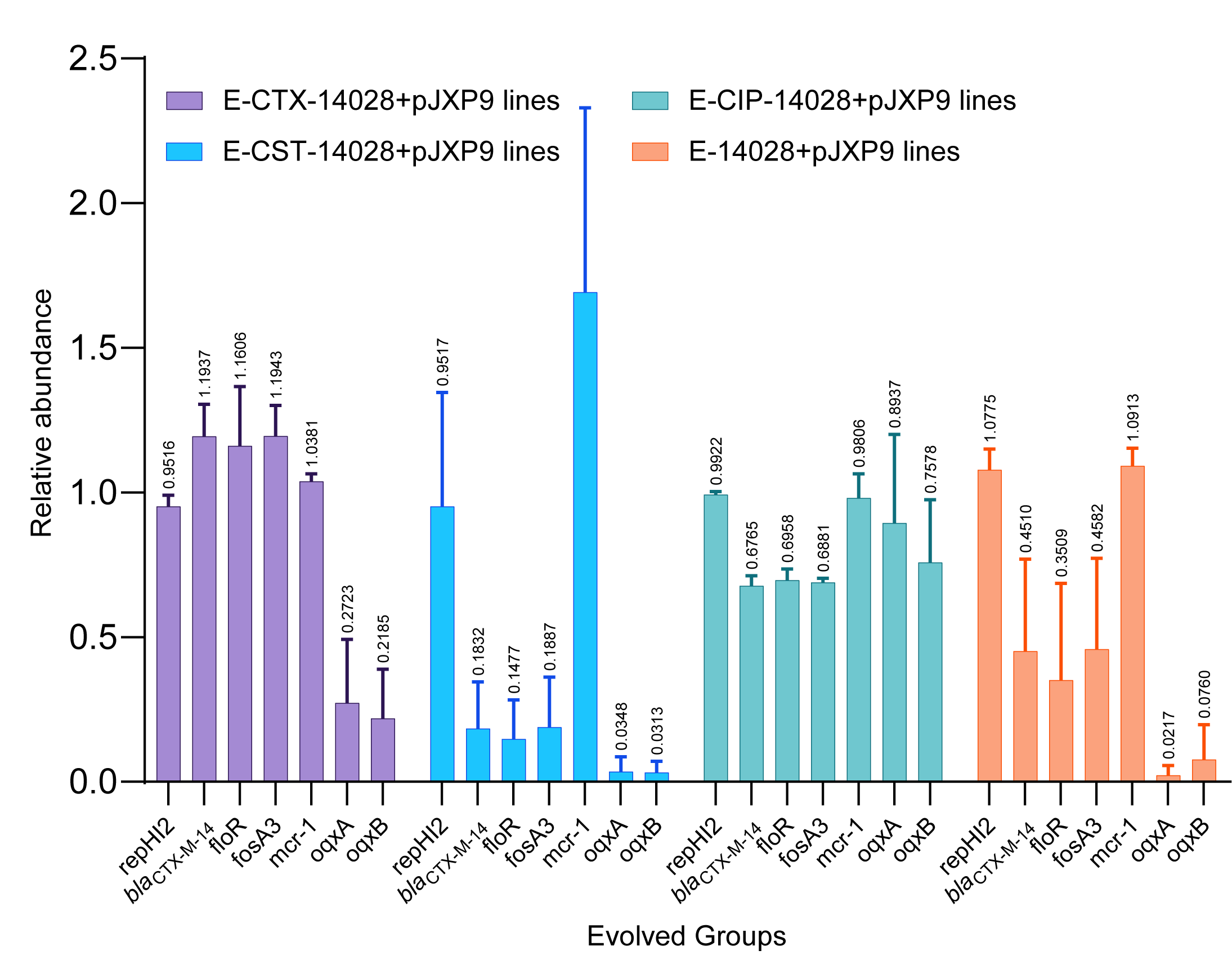

Supplement: FIG S2 [file msystems.00248-22-s0007.tif]

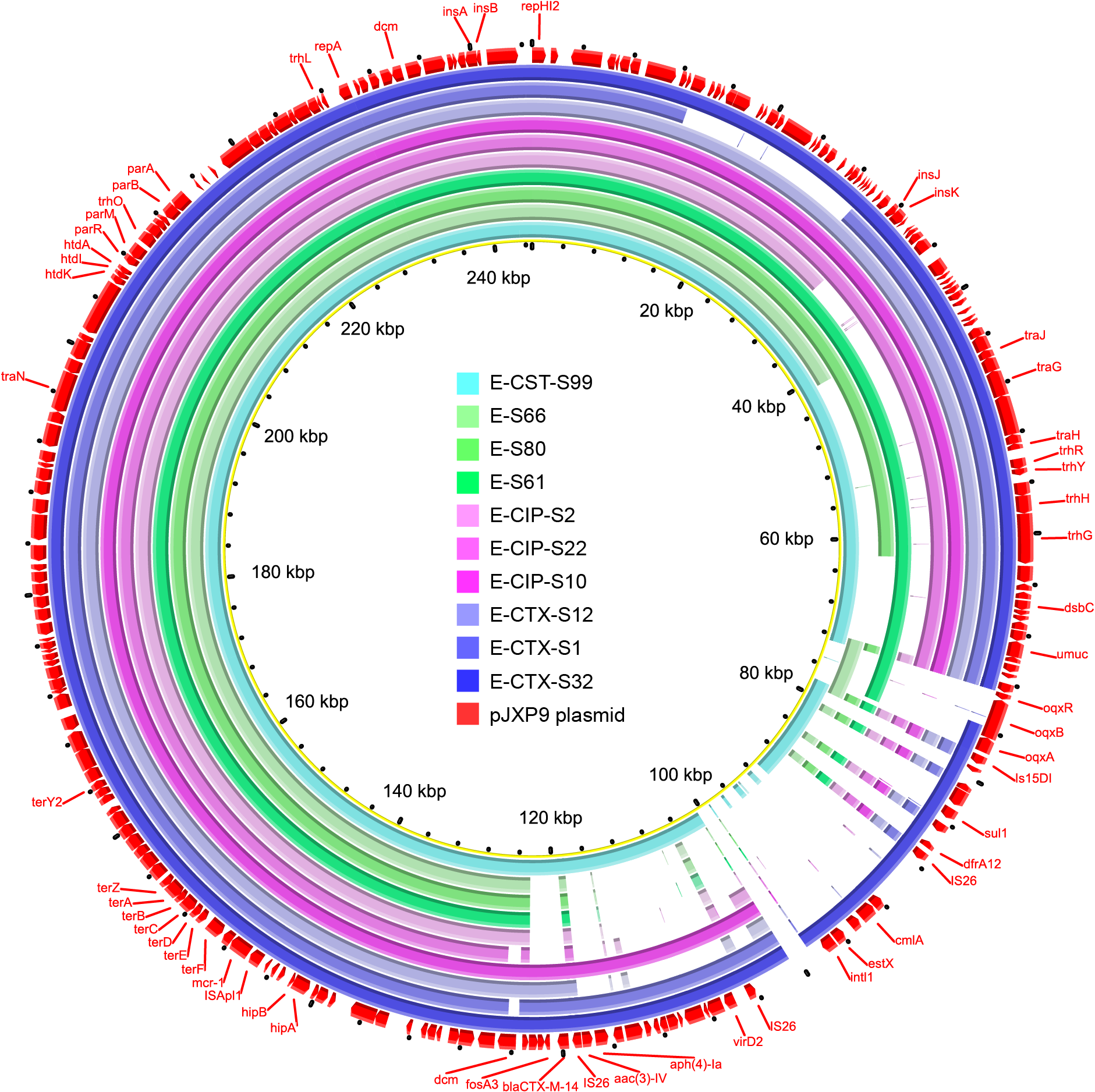

Supplement: FIG S3 [file msystems.00248-22-s0010.tif]

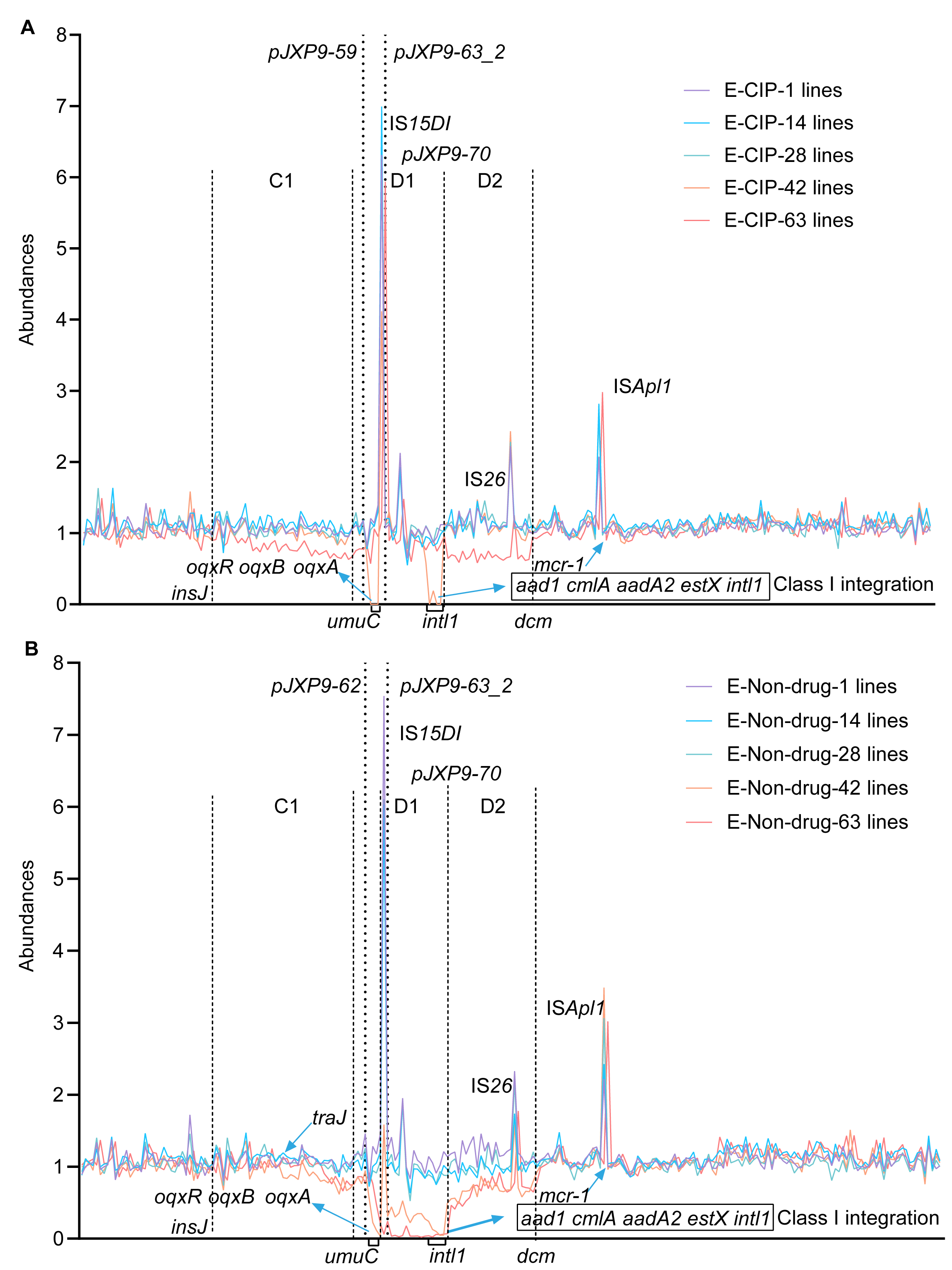

Supplement: FIG S4 [file msystems.00248-22-s0008.tif]

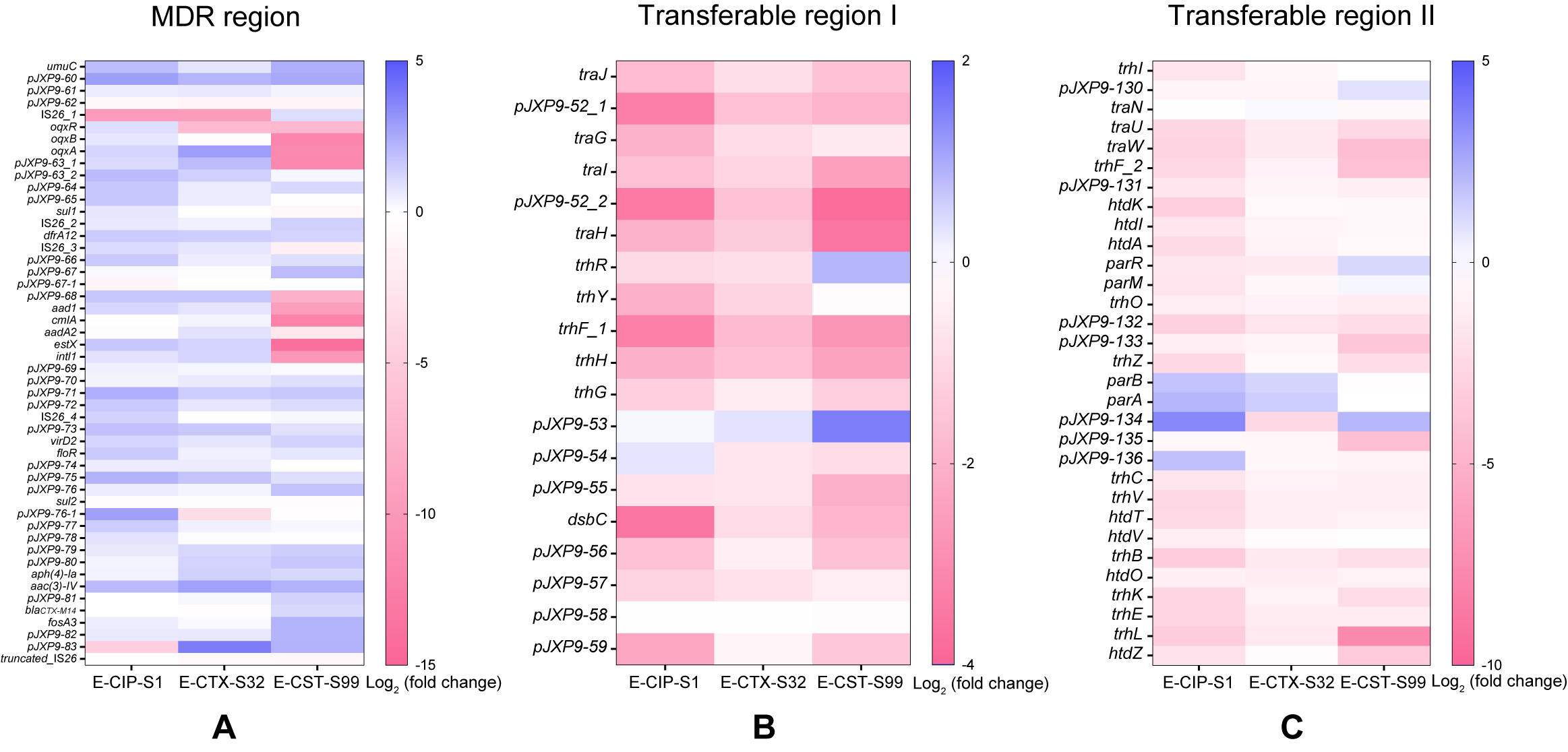

Supplement: FIG S5 [file msystems.00248-22-s0009.tif]

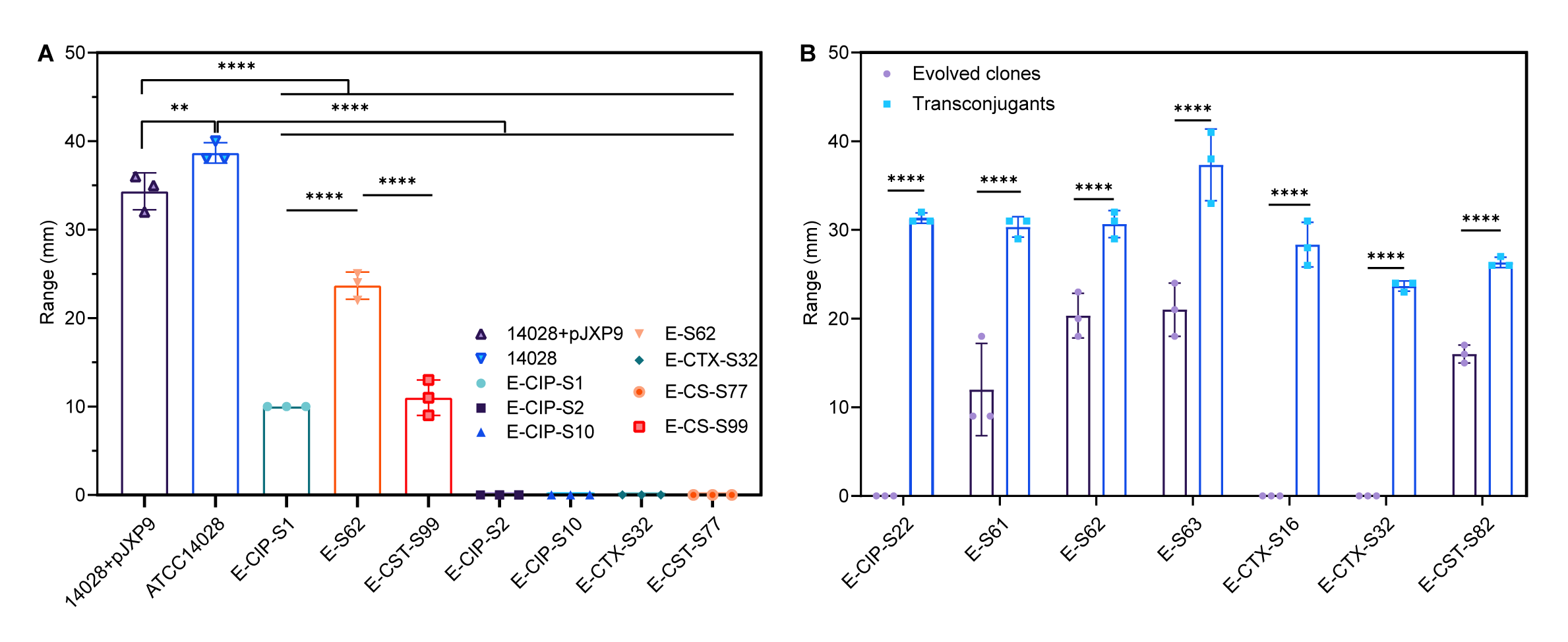

Supplement: FIG S6 [file msystems.00248-22-s0005.tif]
