# Supplementary material for: A Trade-Off for Maintenance of Multidrug-Resistant IncHI2 Plasmids in Salmonella enterica Serovar Typhimurium through Adaptive Evolution
Source: mSystems. 2022 Aug 30;7(5):e00248-22. doi: 10.1128/msystems.00248-22 (PMC9599605; doi:10.1128/msystems.00248-22)
Supplement: TABLE S3 [file msystems.00248-22-s0003.docx]

| Evolved strains (n=240) | | | | Ancestor 14028 carrying pJXP9 (n=1) | | Ancestor 14028 (n=1) |
| --- | --- | --- | --- | --- | --- | --- |
| ARGs (presence or lacking) | Numbers | Tested antibiotic | MICs distribution (μg/mL) | ARGs presence | MICs (μg/mL) | MICs (μg/mL) |
| *mcr-1* (+) | 234 | colistin | 8 | *mcr-1* | 8 | 0.5 |
| *mcr-1* (-) | 6 |  | 0.5 |  |  |  |
| *bla*_CTX-M-14_ (+) | 182 | cefotaxime | >16 | *bla*_CTX-M-14_ | >16 | 0.125 |
| *bla*_CTX-M-14_ (-) | 58 |  | 0.00375 |  |  |  |
| *fosA3* (+) | 181 | fosfomycin | 256-512 | *fosA3* | 512 | 1 |
| *fosA3* (-) | 59 |  | 0.5-1 |  |  |  |
| *oqxAB* (+) | 120 | ciprofloxacin | 0.06 | *oqxAB* | 0.06 | 0.015 |
| *oqxAB* (-) | 120 |  | 0.0075-0.015 |  |  |  |
| *oqxAB* (+) | 120 | nalidixic acid | 16-32 | *oqxAB* | >64 | 4 |
| *oqxAB* (-) | 120 |  | 4 |  |  |  |
| *floR* (+) | 176 | florfenicol | 128-256 | *floR* | 256 | 2 |
| *floR* (-) | 64 |  | 2-4 |  |  |  |
