# Supplementary material for: A Trade-Off for Maintenance of Multidrug-Resistant IncHI2 Plasmids in Salmonella enterica Serovar Typhimurium through Adaptive Evolution
Source: mSystems. 2022 Aug 30;7(5):e00248-22. doi: 10.1128/msystems.00248-22 (PMC9599605; doi:10.1128/msystems.00248-22)
Supplement: TEXT S1 [file msystems.00248-22-s0004.docx]

**Supplementary methods**

**Methods employed for this research are as following:**

**Acquisition of ancestral clones bearing ancestral pJXP9 plasmid and evolved pJXP9 plasmid**

In order to initiate the experimental evolution, conjugation experiments were conducted to acquire ancestral strain bearing pJXP9 plasmid (ATCC14028+pJXP9). Firstly, the plasmid pJXP9 from *S*. Typhimurium JXP9 was transferred to recipient *E. coli* J53 (Azi^R^) by conjugation experiments using the broth-mating method (1) and transconjugants (J53+pJXP9) were selected on selected on Luria-Bertani (LB) plates containing colistin (4 μg/mL) and sodium azide (100 μg/mL). The transconjugant was confirmed by displaying resistance to colistin and sodium azide and carried *repHI2* and *mcr-1* genes, [simultaneously](javascript:;). Then the plasmid pJXP9 from transconjugant J53+pJXP9 was transferred to ancestral strain 14028 by conjugation experiments as described above. As a result, transconjugants were selected from LB plates containing colistin (4 μg/mL) and was confirmed by detecting *invA* gene, which was used as a proxy for the presence of *Salmonella*. The target transconjugants should be *invA* positive and resistant to colistin simultaneously.

To conduct competition experiment among evolved clones and ancestral clones bearing plasmid pJXP9, *lux* reporter system was inserted into ancestral 14028 (sensitive to chloramphenicol) to obtain 14028::*lux.* Briefly, gene cluster *lux*CDABE was linked to the *Pst*I site of pCat-XY (stored in our lab, resistance to chloramphenicol) from *E. coli* WM3064 (cell wall defective strain) resulting in pCat-XY::*lux* using homologous recombination kit ([NEBuilder HiFi DNA Assembly Cloning Kit](https://www.neb.com/products/e5520-nebuilder-hifi-dna-assembly-cloning-kit); New England Biolabs, Beverly, MA USA). Then plasmid pCat-XY::*lux* from *E. coli* WM3064 was transferred to ATCC 14028 by filter mating assays in agar plates containing diaminopimelic acid (DAP, 57 μg/mL). The constructed strains 14028*:lux* were selected on LB plates containing chloramphenicol (25 µg/mL), and were further confirmed using epifluorescence microscopy (Leitz Aristoplan). The obtained 14028:*lux* was then cultivated on LB plates containing sucrose to eliminate pCat-XY plasmid and confirmed on LB plates containing chloramphenicol (25 µg/mL).

To acquire ancestral 14028 bearing the evolved plasmid, the evolved plasmid pJXP9 from end-point evolved 14028::pJXP9 (donor strain) was introduced into constructed 14028::*lux* (recipient strain) using filter mating as described above. Briefly, when grown to log phase in LB broth, donor cultures (evolved clones) and recipient cultures (14028::*lux*) were mixed in a 1:1 ratio, filtered through 0.22-µm pore size membranes (HuanKai Microbial, China) and placed on pre-warmed LB agar plates containing colistin (4 μg/mL). Transconjugants were further verified under a fluorescence microscope. The fluorescent transconjugants harboring evolved plasmid pJXP9 were confirmed by PCR screening for the target gene *mcr-1* (Table. S1).

**S1-PFGE and hybridization analysis**

S1-PFGE was performed to obtain plasmid profiles of 2 ancestral and 20 evolved strains (2). Briefly, whole-cell DNA of these strains embedded in agarose gel plugs was treated with S1 nuclease (Takara, Dalian, China) and separated by PFGE alongside a standard PFGE Marker *Salmonella* *enterica* serotype Braenderup H9812 strain after *Xba*I digestion. Subsequently, Southern blot hybridization was performed with DNA probes specific for *repHI2*, which were non-radioactively labeled with a DIG High Prime DNA labeling and detection kit (Roche Diagnostics, Mannheim, Germany) (3). The *repHI2* probe was generated by PCR using primers *repHI2*-F and *repHI2*-R (Table S1). The pulsed-field gel electrophoresis (PFGE) was performed with the CHEF Mapper XA system (Bio-Rad, Hercules, CA, USA) and hybridizing bands using the reference H9812 marker as a molecular size standard.

**Functional verification of mutant genes**

To confirm the impact of chromosomal gene mutation on the fitness cost of pJXP9 plasmid carriage, the selected target genes were knocked out in the ancestral 14028 bearing plasmid pJXP9 (14028+pJXP9) /evolved plasmid pJXP9 (14028+pJXP9, three types of plasmids) using a two-plasmid system (pCaspa-pSGKp) (4). Briefly, sgRNAs flanked by *Bam*HI and *Xba*I restriction sites specific for the selected target genes *ahpC*, *osmY* and *ybgS* were designed as previously described (5). Then targeted sgRNAs were digested with *Bam*HI and *Xba*I and then were ligated into the pSGKp containing *sacB* and *arr-3*. At the same time, modified pCaspa containing *Cas9* and *TetA* genes was transferred to 14028+pJXP9 and 14028+pJXP9 by filter mating assays and were followed by selection on LB agar containing [tetracycline](javascript:;) (100 μg/mL) (6). Transconjugants were selected and inoculated in 100 mL glucose-free LB broth at 30°C with shaking (200 rpm). After incubation to OD_600_ = 0.2, arabinose was added to 0.1% and the cells were prepared for electroporation at OD_600_=0.7 as previously described (4). pSGKp containing the targeted sgRNAs was electroporated into the cells and electroporants were selected on LB agar containing [tetracycline](javascript:;) (100 μg/mL) and rifampin (50 μg/mL). The electroporants were further streaked onto LB agar containing 5 % sucrose and incubated at 37°C overnight and then selected on plates containing rifampin or [tetracycline](javascript:;) to confirm the successful self-curing of pSGKP or pCaspa, respectively. The N20 - base targeting deleted genes and detection primers are listed in Table S2.

**Swimming motility experiments**

Bacterial swimming motility was assessed using LB plates containing 0.25 % agar as previously described (7). In brief, 2 μL overnight bacteria were suspended to OD_600_ = 0.5 and added to the center of the plates that were then incubated at 37°C 6 h. The area of the bacterial swimming from the inoculated spot was measured using a caliper. Individual values of at least 3 independent experiments.

**REFERENCES**

1. Sun J, Yang RS, Zhang Q, Feng Y, Fang LX, Xia J, Li L, Lv XY, Duan JH, Liao XP, Liu YH. 2016. Co-transfer of blaNDM-5 and mcr-1 by an IncX3-X4 hybrid plasmid in Escherichia coli. Nat Microbiol 1:16176.

2. Li R, Du P, Zhang P, Li Y, Yang X, Wang Z, Wang J, Bai L. 2021. Comprehensive Genomic Investigation of Coevolution of mcr genes in Escherichia coli Strains via Nanopore Sequencing. Glob Chall 5:2000014.

3. Li R, Xie M, Zhang J, Yang Z, Liu L, Liu X, Zheng Z, Chan EW, Chen S. 2017. Genetic characterization of mcr-1-bearing plasmids to depict molecular mechanisms underlying dissemination of the colistin resistance determinant. J Antimicrob Chemother 72:393-401.

4. Anonymous. 2018. CRISPR-Cas9 and CRISPR-Assisted Cytidine Deaminase Enable Precise and Efficient Genome Editing in Klebsiella pneumoniae.

5. He YZ, Yan JR, He B, Ren H, Kuang X, Long TF, Chen CP, Liao XP, Liu YH, Sun J. 2021. A transposon-associated CRISPR/Cas9 system specifically eliminates both chromosomal and plasmid-borne mcr-1 in Escherichia coli. Antimicrob Agents Chemother doi:10.1128/AAC.01054-21:AAC0105421.

6. Chen W, Zhang Y, Zhang Y, Pi Y, Gu T, Song L, Wang Y, Ji Q. 2018. CRISPR/Cas9-based Genome Editing in Pseudomonas aeruginosa and Cytidine Deaminase-Mediated Base Editing in Pseudomonas Species. iScience 6:222-231.

7. Liu Y, Tong Z, Shi J, Jia Y, Deng T, Wang Z. 2021. Reversion of antibiotic resistance in multidrug-resistant pathogens using non-antibiotic pharmaceutical benzydamine. Commun Biol 4:1328.
