# Supplementary material for: A Trade-Off for Maintenance of Multidrug-Resistant IncHI2 Plasmids in Salmonella enterica Serovar Typhimurium through Adaptive Evolution
Source: mSystems. 2022 Aug 30;7(5):e00248-22. doi: 10.1128/msystems.00248-22 (PMC9599605; doi:10.1128/msystems.00248-22)
Supplement: TABLE S1 [file msystems.00248-22-s0001.docx]

| **Categories** | **Target** | **Primers** | **Nucleotide sequences**  **(5’ to 3’)** | **Amplicon size (bp)** | **References** |
| --- | --- | --- | --- | --- | --- |
| **qPCR primers** | **16srRNA** | RT-16S-F  RT-16S-R | ATTAGATACCCTGGTAGTCCACGC TTGCGGGACTTAACCCAAC | 316 | (1) |
|  | ***repHI2*** | RT-repHI2-F  RT-repHI2-R | GTAACCACTAAATACCCGGG  TTCCTGGTTTCGGTTTAGCC | 261 | (2) |
|  | ***mcr-1*** | RT-mcr-1-F  RT-mcr-1-R | ACACTTATGGCACGGTCTATG  GCACACCCAAACCAATGATAC | 120 | (2) |
|  | ***oqxB*** | RT-oqxB-F  RT-oqxB-R | TCCTGATCTCCATTAACGCCCA  ACCGGAACCCATCTCGATGC | 131 | (3) |
|  | ***bla*_CTX-M-14_** | RT-*bla*_CTX-M-14_-F  RT-*bla*_CTX-M-14_-R | TCAAGCCTGCCGATCTGGTTAAC  CAGCGTAGGTTCAGTGCGATCC | 218 | This study |
| **PCR primers** | ***repHI2*** | repHI2-F  repHI2-R | TTTCTCCTGAGTCACCTGTTAACAC  GGCTCACTACCGTTGTCATCCT | 644 | (4) |
|  | ***mcr-1*** | mcr-1-F  mcr-1-R | GGGTGTGCTACCAAGTTTGC  CATTGGCGTGATGCCAGTTT | 1126 | (5) |
|  | ***oqxB*** | oqxB-F  oqxB-R | CGAAGAAAGACCTCCCTACCC  CGCCGCCAATGAGATACA | 240 | (6) |
|  | ***bla*_CTX-M-14_** | *bla*_CTX-M14_-F  *bla*_CTX-M14_-R | TTTGCGATGTGCAGTACCAGTAA  CGATATCGTTGGTGGTGCCATA | 544 | (7) |
|  | ***fosA3*** | fosA3-F  fosA3-R | GCGTCAAGCCTGGCATTT  GCCGTCAGGGTCGAGAAA | 282 | (8) |
|  | ***floR*** | floR-F  floR-R | GCGATATTCATTACTTTGGC  TAGGATGAAGGTGAGGAATG | 425 | (9) |
|  | ***invA*** | invA-F  invA-R | GTGAAATTATCGCCACGTTCGGGCAA  TCATCGCACCGTCAAAGGAACC | 284 | (10) |
| **Test for gene deletions** | ***ahpC*** | a-testF  a-testR | AGGTTTGGTCAGTTTCTCAAGGTAA  AGCCTTTGTTAAGGTAATGTAGAGCG | _ | This study |
|  | ***osmY*** | *o-testF*  *o-testR* | ATGTCGACCGCTAAATATGAGC  CTTCGTACTTTCCCTGCCTT | _ | This study |
|  | ***ybgS*** | *y-testF*  *y-testR* | CTGACACGTGCACTGTTGGA  ATGAGAAGGGTAATGACCACCAA | _ | This study |
| **20-nt base for guiding Cas9** | ***ahpC*** | *ahpC-sgRNA* | TCGCCTTCTTTCCATTTCGC | _ | This study |
|  | ***osmY*** | *osmY-sgRNA* | TCCACTTTCACTTTACGGGA | _ | This study |
|  | ***ybgS*** | *ybgS-sgRNA* | TCTAACGCGCAATCCAGTAA | _ | This study |

**References**

1. Chen C, Cui C-Y, Yu J-J, He Q, Wu X-T, He Y-Z, Liu Y-H. 2020. Genetic diversity and characteristics of high-level tigecycline resistance Tet(X) in Acinetobacter species. Genome Med 12:111.

2. Kieffer N, Royer G, Decousser JW, Bourrel AS, Palmieri M, Ortiz De La Rosa JM, Jacquier H, Denamur E, Nordmann P, Poirel L. 2019. mcr-9, an Inducible Gene Encoding an Acquired Phosphoethanolamine Transferase in Escherichia coli, and Its Origin. Antimicrob Agents Chemother 63:e00965-19.

3. Kim HB, Wang M, Park CH, Kim EC, Jacoby GA, Hooper DC. 2009. oqxAB encoding a multidrug efflux pump in human clinical isolates of Enterobacteriaceae. Antimicrob Agents Chemother 53:3582-4.

4. Carattolia A, Bertinia A, Villaa L, Falbob V, Hopkinsc KL, Threlfallc EJ. 2005. Identification of plasmids by PCR-based replicon typing. J Microbiol Methods 63:219-28.

5. R. H, A. K, Y. A, S. H. 2018. Detection of plasmid-mediated colistin-resistant and carbapenem-resistant genes by multiplex PCR. MethodsX 5:532-6.

6. Chen X, Zhang W, Pan W, Yin J, Pan Z, Gao S, Jiao X. 2012. Prevalence of qnr, aac(6')-Ib-cr, qepA, and oqxAB in Escherichia coli isolates from humans, animals, and the environment. Antimicrob Agents Chemother 56:3423-7.

7. Edelstein M, Pimkin M, Palagin I, Edelstein I, Stratchounski L. 2003. Prevalence and molecular epidemiology of CTX-M extended-spectrum beta-lactamase-producing Escherichia coli and Klebsiella pneumoniae in Russian hospitals. Antimicrob Agents Chemother 47:3724-32.

8. Hou J, Huang X, Deng Y, He L, Yang T, Zeng Z, Chen Z, Liu JH. 2012. Dissemination of the fosfomycin resistance gene fosA3 with CTX-M beta-lactamase genes and rmtB carried on IncFII plasmids among Escherichia coli isolates from pets in China. Antimicrob Agents Chemother 56:2135-8.

9. Faldynova M, Pravcova M, Sisak F, Havlickova H, Kolackova I, Cizek A, Karpiskova R, Rychlik I. 2003. Evolution of antibiotic resistance in Salmonella enterica serovar typhimurium strains isolated in the Czech Republic between 1984 and 2002. Antimicrob Agents Chemother 47:2002-5.

10. Malorny B, Hoorfar J, Bunge C, Helmuth R. 2003. Multicenter validation of the analytical accuracy of Salmonella PCR: towards an international standard. Appl Environ Microbiol 69:290-6.
