# Supplementary material for: A Trade-Off for Maintenance of Multidrug-Resistant IncHI2 Plasmids in Salmonella enterica Serovar Typhimurium through Adaptive Evolution
Source: mSystems. 2022 Aug 30;7(5):e00248-22. doi: 10.1128/msystems.00248-22 (PMC9599605; doi:10.1128/msystems.00248-22)
Supplement: TABLE S2 [file msystems.00248-22-s0002.docx]

| Evolved clones | Clean data (Q30, %) | Assembled contigs | Largest contig | Total length | GC (%) | N50 | L50 | N's per 100 kbp | Average Coverage |
| --- | --- | --- | --- | --- | --- | --- | --- | --- | --- |
| E-CIP-S1 | 98.0465 | 113 | 449622 | 4792051 | 52.17 | 131752 | 10 | 0 | 321 |
| E-CIP-S2 | 98.3738 | 121 | 401625 | 4793662 | 52.16 | 95529 | 10 | 0 | 297 |
| E-CIP-S10 | 98.1216 | 69 | 622466 | 4795640 | 52.16 | 150065 | 8 | 0 | 305 |
| E-CIP-S11 | 98.1341 | 68 | 542311 | 4794937 | 52.17 | 156005 | 7 | 0 | 318 |
| E-CIP-S22 | 98.1124 | 121 | 400520 | 4794339 | 52.16 | 102202 | 11 | 0 | 293 |
| E-S61 | 98.2182 | 70 | 622466 | 4794980 | 52.16 | 209049 | 6 | 0 | 307 |
| E-S62 | 97.8973 | 66 | 622466 | 4789110 | 52.17 | 209049 | 6 | 0 | 308 |
| E-S63 | 97.9999 | 69 | 536598 | 4795227 | 52.16 | 192218 | 7 | 0 | 311 |
| E-S66 | 98.2359 | 72 | 596774 | 4796363 | 52.16 | 156194 | 8 | 0 | 329 |
| E-S80 | 98.0501 | 71 | 622466 | 4792810 | 52.16 | 209049 | 7 | 0 | 264 |
| E-CTX-S1 | 97.8328 | 68 | 622466 | 4795350 | 52.17 | 156194 | 7 | 0 | 260 |
| E-CTX-S12 | 97.7864 | 69 | 622466 | 4764440 | 52.16 | 183627 | 7 | 0 | 257 |
| E-CTX-S16 | 98.1256 | 65 | 622466 | 4763383 | 52.16 | 225811 | 6 | 0 | 214 |
| E-CTX-S17 | 97.8518 | 67 | 622466 | 4763887 | 52.16 | 209049 | 7 | 0 | 240 |
| E-CTX-S32 | 97.8003 | 72 | 547323 | 4796173 | 52.16 | 148499 | 10 | 0 | 209 |
| E-CST-S72 | 97.7703 | 66 | 622466 | 4733707 | 52.2 | 225811 | 6 | 0 | 262 |
| E-CST-S74 | 98.0828 | 69 | 572172 | 4734135 | 52.2 | 225811 | 6 | 0 | 225 |
| E-CST-S77 | 97.8801 | 66 | 622466 | 4728008 | 52.2 | 209049 | 7 | 0 | 230 |
| E-CST-S82 | 97.9396 | 71 | 439015 | 4794835 | 52.16 | 150065 | 9 | 0 | 240 |
| E-CST-S99 | 97.7192 | 72 | 294233 | 4796085 | 52.16 | 148498 | 13 | 0 | 205 |
